# The immune response modulated by inoculation of commensal bacteria at birth impacts the gut microbiota and prevents *Salmonella* colonization

**DOI:** 10.1080/19490976.2025.2474151

**Published:** 2025-03-13

**Authors:** Florent Kempf, Rosanna Drumo, Anne Marie Chaussé, Pierrette Menanteau, Tereza Kubasova, Sylvie Roche, Anne Christine Lalmanach, Rodrigo Guabiraba, Thierry Chaumeil, Guillaume Larivière-Gauthier, Ignacio Caballero-Posadas, Béatrice Laroche, Ivan Rychlík, Isabelle Virlogeux-Payant, Philippe Velge

**Affiliations:** a ISP, INRAE, Université François Rabelais de Tours, Nouzilly, France; b Department of Microbiology and antimicrobial resistance, Veterinary Research Institute, Brno, Czech Republic; c Plate-Forme d’Infectiologie Expérimentale, INRAE, Nouzilly, France; d MaIAGE, INRAE, Jouy-en-Josas, France

**Keywords:** Salmonella, carrier-state, excretion, chicken, super-shedder, immune response, microbiota, virulence

## Abstract

Super- and low-shedding phenomena have been observed in genetically homogeneous hosts infected by a single bacterial strain. To decipher the mechanisms underlying these phenotypes, we conducted an experiment with chicks infected with *Salmonella* Enteritidis in a non-sterile isolator, which prevents bacterial transmission between animals while allowing the development of the gut microbiota. We investigated the impact of four commensal bacteria called Mix4, inoculated at hatching, on chicken systemic immune response and intestinal microbiota composition and functions, before and after *Salmonella* infection. Our results revealed that these phenotypes were not linked to changes in cell invasion capacity of bacteria during infection. Mix4 inoculation had both short- and long-term effects on immune response and microbiota and promoted the low-shedder phenotype. Kinetic analysis revealed that Mix4 activated immune response from day 4, which modified the microbiota on day 6. This change promotes a more fermentative microbiota, using the aromatic compounds degradation pathway, which inhibited *Salmonella* colonization by day 11 and beyond. In contrast, control animals exhibited a delayed TNF-driven pro-inflammatory response and developed a microbiota using anaerobic respiration, which facilitates *Salmonella* colonization and growth. This strategy offers promising opportunities to strengthen the barrier effect against *Salmonella* and possibly other pathogens.

## GRAPHICAL ABSTRACT

**Figure:**
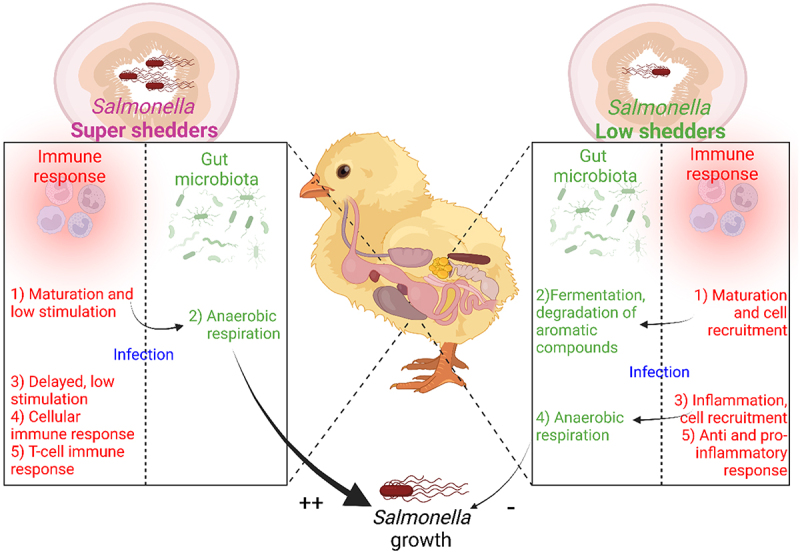

## Introduction

*Salmonella* infections are of significant medical and economic concern in developed countries. In 2021, the EU reported 15.3 cases per 100 000 population, an increase from 2020 but still below pre-COVID-19 pandemic levels.^1^ The proportion of hospitalized cases was 38.9% with a fatality rate of 0.22%. According to the CDC, *Salmonella* is also a major cause of death and hospitalization in the USA and according to WHO, it is still considered a priority bacterial pathogen in 2024 on every continent.^2^ *Salmonella* Enteritidis (SE) represents the predominant serovar in Europe, associated with 67.3% of all *Salmonella* outbreaks.^1^ These foodborne outbreaks are mainly related to poultry product consumption, in particular through the ingestion of contaminated eggs or poultry meat.^3^ In both chickens and pigs, *Salmonella* can colonize the intestine, then translocate to induce a systemic infection, sometimes leading to a lethal systemic infection, or more generally to a long-term asymptomatic carrier state.^4^

*Salmonella*-infected animals often present highly heterogeneous shedding levels. The same phenomenon may arise in other infectious diseases.^5^ The infected individuals that harbor and shed a given pathogen at higher concentrations than their congeners are commonly referred to as “super-shedders.” As they present a higher transmission rate, epidemiological modeling has shown that 20% of the individuals presenting the highest shedding levels are involved in 80% of the transmission events. Thus, super-shedders are a key target of control strategies; subsequently, strategies that aim at identifying and controlling these animals are required.^5,6^

Several causes may explain the super-shedding phenomenon. Strain-level genetic differences have been reported in the case of enterohemorrhagic *Escherichia coli* O157:H7^7^ or *Campylobacter jejuni*.^8^ Host-related factors may include differential immune responses to *Salmonella* at a transcriptional level^9,10^ between susceptible and resistant chicken lines.^11,12^ However, *Salmonella* heterogeneous shedding has been observed even in the context of a genetically homogeneous infected host population (e.g. inbred mice^13^ and chickens).^14,15^ Besides, several studies have investigated the microbiota–pathogen interactions and their role in the establishment of *Salmonella* super-shedding.^16,17^ In chicken, we demonstrated that (1) in axenic chicken, *Salmonella* highly colonizes the gut leading to the super-shedding phenotype; (2) inoculating microbiota of hens to chicks before infection completely inhibit *Salmonella* colonization; (3) modulating the gut microbiota (GM) by means of antibiotics subsequently modifies the patterns of *Salmonella* shedding levels; and (4) the presence of specific taxa before infection determines the acquisition of the super- or low-shedder phenotypes.^18^

All these results emphasized the importance of the GM in the low- or super-shedder phenotype and suggested the possibility to manipulate the GM to limit *Salmonella* colonization and consequently reduce their fecal shedding. In line with this, we have already demonstrated that a consortium of four commensal bacteria (namely ‘Mix4’) inoculated before infection successfully reduces the *Salmonella* shedding levels.^18^ Likewise, histological observations revealed that the Mix4 inoculation is involved in the maturation of the gut adaptive immune system. The role played by commensal symbiotic bacteria in the maturation of the gut immune system has been recognized for long in mammalian hosts.^19^ Similarly, in chickens, it has been shown that early microbial colonization of the intestine is required for the proper development of the intestinal immune system, in particular adaptive responses.^20,21^

In order to predict the animal susceptibility and counterbalance the ability of pathogens to overcome the barrier effect in some animals, it is important to better understand the causes of heterogeneity of infection. In this work, we aimed to decipher the causes leading to the low- and super-shedder phenotypes by testing the possible microevolution of *Salmonella* virulence during infection, and by analyzing the interactions between the immune response and GM development before and after *Salmonella* infection. For this purpose, we used a specific isolator rearing system that minimizes cross-contamination^15,22^, and allows us to control for several factors likely to be involved in the appearance of super-shedding such as dose, bacterial strain, host genetics, and bacterial transmission. Within this framework, we studied (1) the virulence traits of *Salmonella* strains recovered from low- and super-shedding chickens and (2) the effects of *Salmonella* infection and Mix4 inoculation on the kinetics of GM composition and putative functions, and expression of systemic immunity genes.

## Results

### The inoculation of four commensal bacteria (Mix4) has a protective effect against S. Enteritidis infection

**Figure 1.**
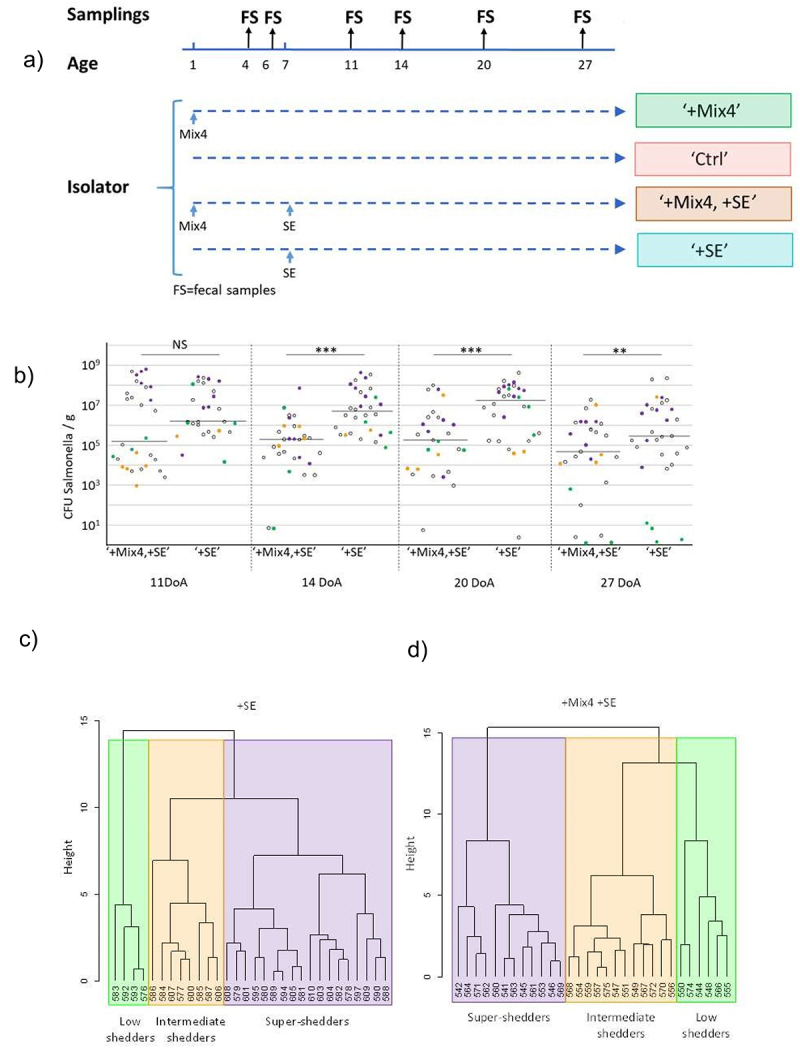
Experimental infection of chickens by *Salmonella* Enteritidis in the presence of a cocktail of four commensal bacteria. (a) The 140 specific-pathogen-free chicks were splatted in four isolators and orally inoculated (‘Mix4’) or not (‘Ctrl’) at 1 days of age (DoA) with the four commensal bacteria. Then there were orally inoculated with 5 × 10^4^ *salmonella* enteritidis (SE) at 7 DoA. Fresh fecal samples (FS) were collected at 4, 6, 11, 14, 20, 27 DoA. The fecal samples recovered were split in two part: one part was immediately frozen in liquid nitrogen for DNA extraction and 16S rRNA gene sequencing and the second part was used to quantify *salmonella* load (b) levels of *Salmonella* excretion for the isolator ‘+Mix4+SE’ and ‘+SE’ at 11, 14, 20, 27 DoA. The chicks kept for 16S metabarcoding are represented by colored solid dots according to the shedding category (green for low shedders, orange for intermediate shedders, purple for super shedders; 15 and 14 chickens for isolator ‘+Mix4+SE’ and ‘+SE’ respectively); the other chicks are represented by empty dots. (c) and (d) hierarchical clustering based on the level of *salmonella* excretion, defining the shedding categories of the chicken bred in isolator ‘+Mix4+SE’ (C) and ‘+SE’ (D). Three clusters of chickens were considered in this study: low, intermediate and super shedders (LS, IS, SS; in green, orange and purple respectively).

To investigate the role of four commensal bacteria (referred to as Mix4) consisting of strains of *E. coli*, *Lactobacillus rhamnosus*, *Enterococcus faecium* and *Clostridium butyricum*, on the low- and super-shedder phenotypes of *Salmonella*, 140 chicks were reared in four separate isolators after oral administration of the Mix4 or of PBS after hatch and were infected or not with *S*. Enteritidis at 7 days of age (Figure 1(a)). We chose to infect chicks in isolators, because this setup significantly reduces animal re-infection and host-to-host transmission of *Salmonella*. This reduction allowed us to more clearly observe the heterogeneity of *Salmonella* infection and the emergence of the low- and super-shedder phenotypes.^15,22^ This heterogeneity of infection was observed in fresh fecal samples collected throughout the kinetics and was characterized by shedding levels ranging from no *Salmonella* to more than 1 × 10^8^ *Salmonella*/g feces (Figure 1(b)) as already described.^18^ When we compared the median level of colonization, we observed a significant reduction between animals infected by *S*. Enteritidis and those that received the Mix4 before *Salmonella* inoculation (‘+SE’ vs ‘+Mix4 +SE’). These differences reached 2 logs 10 of difference at 13 days after infection (*p* < 0.001) (Figure 1(b)). As previously described, chicks could be clustered in three main groups with a hierarchical clustering of *Salmonella* shedding kinetics levels as illustrated in Figure 1(c,d).^15^ This clustering included 30 ‘+SE’ chicks and ‘+Mix4+SE’ chicks. The first group, represented in green, consisted of chicks with the lowest levels of *Salmonella* and was designated as “low-shedders” (LS). The second group, in purple, included chicks with very high levels of fecal excretion and was designated here as “super-shedder” (SS). The third group, in orange, exhibited *Salmonella* levels between those of the low or the super-shedder groups and was classified as “intermediate shedders” (IS).

### Strains isolated from LS and SS did not present differences in their virulence traits

**Figure 2.**
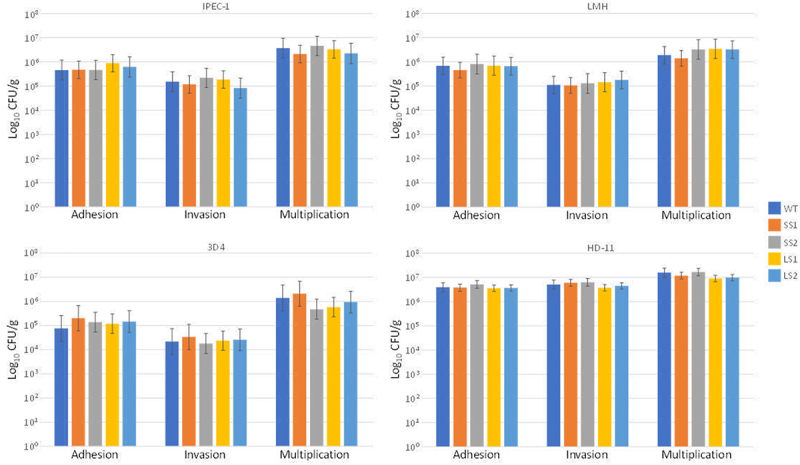
*In vitro* virulence of *Salmonella* enteritidis strains recovered before and after chick infection. Two strains were recovered from fecal samples of low-shedders at the end of the experiment (LS1 and LS2), and two strains from the super-shedders (SS1 and SS2). Adhesion, invasion and intracellular multiplication capabilities of these strains were compared to the parental strain (WT) inoculated to chicks with the gentamicin protective assay at a multiplicity of infection of 10 bacteria for 1 cell. Virulence was tested in chicken (LMH) and porcine (IPEC-1) epithelial cell lines, and in chicken (HD11) and porcine macrophage (3D4) cell lines. Results are expressed as mean ± SEM of the number of bacteria per 10^7^ CFU deposited on the cells. Experiments were in duplicate and repeated at least three times for each strain.

One hypothesis for the development of the LS and SS phenotypes might be that *Salmonella* virulence can change during infection of a host. To test this hypothesis, two *Salmonella* colonies were recovered from fecal samples of two LS animals and two *Salmonella* colonies from two SS animals at the end of the experiment. Then, we compared adhesion, invasion, and intracellular multiplication capabilities of these strains to those of the inoculated strain using the gentamicin protection assay, as previously described^23^. Putative virulence modifications were tested in LMH and IPEC-1, chicken and porcine epithelial cell lines, respectively, and in HD11 and 3D4, chicken and porcine macrophage cell lines, respectively. However, no significant differences were observed for any criterion, regardless of the cell line used (Figure 2). These results strongly suggest that the appearance of the LS and SS phenotypes is not linked to changes in the cell adhesion, invasion, and intracellular multiplication capacity of the *S*. Enteritidis strain during *in vivo* infection, although this does not rule out other modifications, such as in its metabolisms.

### Gut microbiota overall diversity

Fresh fecal samples were collected individually from all chicks before (at 4 and 6 days of age) and after infection (at 11, 14, 20, 27 days of age, Figure 1(a)). The GM composition was analyzed kinetically in the ‘+Mix4+SE’ and the ‘+SE’ chicks infected with *Salmonella* (shown in color in Figure 1(b)), including an equivalent number of the three shedding level categories shown in Figures 1(c,d). Their GM composition was compared with that of uninfected control chicks inoculated with (‘+Mix4’,) or without the Mix4 (‘Ctrl’). A total of 241 chicks could be analyzed: 40 ‘+Mix4’, 42 ‘Ctrl’, 84 ‘+Mix4+SE’ and 75 ‘+SE’ chicks.

**Figure 3.**
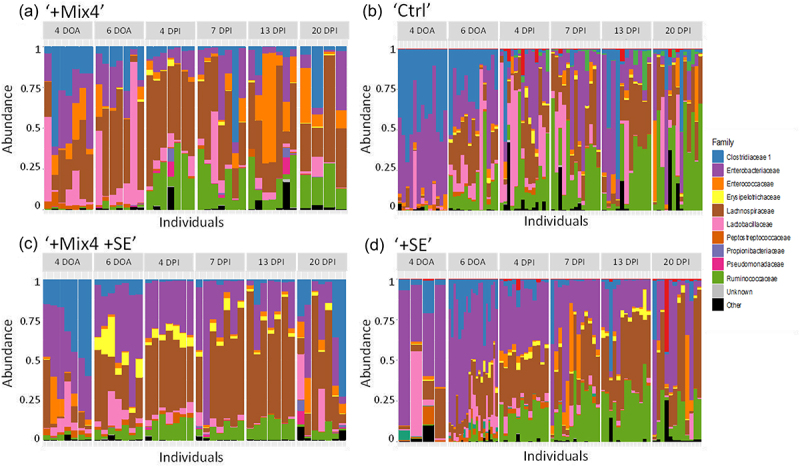
Bacterial family-level composition. Bar plots show relative abundance (%) of the top 10 bacterial genera in fecal samples from chicks reared in the isolator ‘+Mix4’ (a), ‘Ctrl’ (b), ‘+Mix4+SE’ (c), and ‘+SE’ (d), and for each age category before (i.e. 4, 6 days of age) and after infection by S. Enteritidis (i.e. 4, 7, 13 and 20 days of age). The “other” in the figure represents bacterial families, identified but not present in the 10 most abundant families.

A large part of the diversity was related to the Proteobacteria because abundances of Firmicutes remained high but stable throughout the experiment. At the family level, a trend was observed in the four isolators with Enterobacteriaceae dominating Proteobacteria (Figure 3). In the ‘+SE’ isolator, Enterobacteriaceae accounted for 33.9% of the taxa at the end of the experiment, irrespective of the presence of *Salmonella* (<1%). These results are in line with previous studies showing a decrease in Enterobacteriaceae over time during the development of the chicken GM.^24^ Similarly, Clostridiaceae abundance decreased during the experiment in all isolators (e.g. from 23.8% on day 4 to 11.5% on day 27 in isolator ‘+Mix4’). In contrast, the abundance of Ruminococcaceae and Lachnospiraceae increased in all isolators. Lachnospiraceae became predominant in the non-infected isolators (37.3%, 32.3% of the total abundance for isolator ‘+Mix4’ and ‘Ctrl’, respectively) and the Ruminococcaceae dominated in isolator ‘+Mix4+SE’ (32.7%).

While all isolators show a similar abundance of Enterobacteriaceae, striking differences emerged at the genus level. In particular, *Klebsiella* sp. was highly abundant in the ‘+SE’ isolator representing 23.4% at 11 DoA but decreasing over time to represent 3.3% at 27 DoA. Among the other isolators, *Escherichia* was dominant and *Klebsiella* sp. remained below 3.5% at all-time points. These differences likely reflect the role of environment on the initial bacterial implantation in the gut microbiota, while retaining the same physiological functions.^25^ It should be noted that these isolator-related differences decreased over the course of the experiment, confirming that the non-sterile isolators did not hamper the development of a complex and diverse microbiota as previously described.^15^

### *Impact of Mix4 and* Salmonella *Enteritidis inoculations on gut microbiota α-diversity*

To analyze the effect of Mix4 inoculation and *Salmonella* infection on GM composition, we scrutinized the GM diversity using two α-diversity indices (Chao1 and Shannon indexes, Supplementary Figure S1). Strong significant differences between age categories were observed using the Chao1 index (*p* < 0.001), indicating an increase in bacterial richness over time. In contrast, the Shannon diversity index demonstrated no significant differences between the isolators, despite a slight increase in values (e.g. from H = 2.04 ± 0.28 at 4 days of age to H = 2.75 ± 0.18 at 27 days of age in the ‘+Mix4’isolator). These results indicate that, while the overall number of OTUs increased over time, reflecting the gradual colonization of GM by environmental bacteria, the GM was constantly dominated by a few abundant OTUs at all time-points of the experiment. Moreover, when comparing α-diversity between different isolators at the same time points, we observed only a few significant differences. These results confirm that, despite minor differences in the GM development, chickens, in all isolators, tend to acquire such a complex and rich GM. Furthermore, the similar α-diversity patterns suggest that neither *Salmonella* infection nor Mix4 inoculation significantly modified the species richness and diversity of the GM. Alternatively, this result showed that variation in α-diversity cannot explain, in our study, the drop of *Salmonella* colonization.

### *Impact of Mix4 and* Salmonella *Enteritidis inoculations on the composition of microbiota and its putative functions*

**Figure 4.**
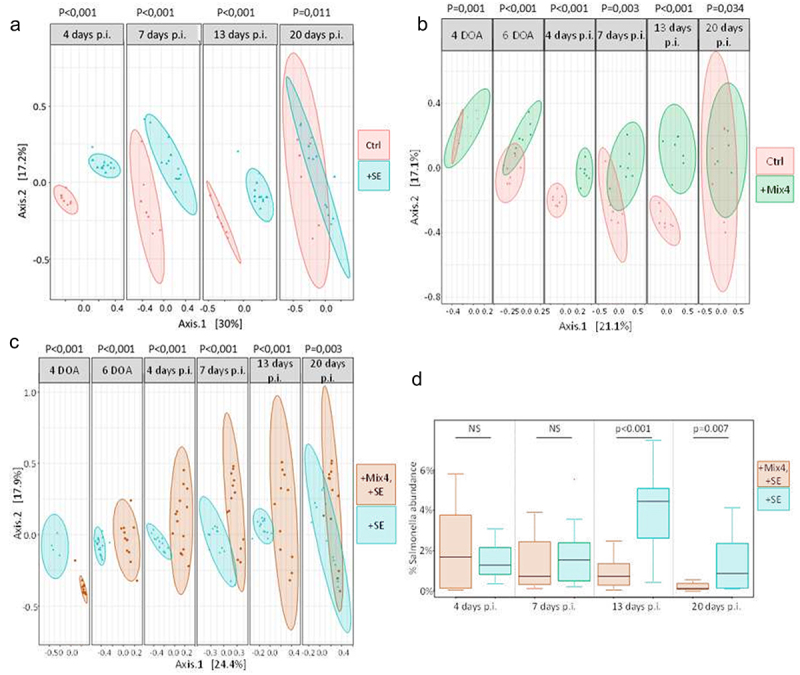
Principal coordinate analysis summarizing Bray-Curtis distances between chicks. (a), (b), (c), the bray-curtis distances were assessed between the four isolators: ‘Ctrl’, ‘+Mix4’, ‘+SE’ and ‘+Mix4+SE’ at each age category, before infection (days of age) and after infection (days post infection). In non-infected isolators, 35 chicks were reared to 27 days of age without being exposed to any inoculation (‘Ctrl’) or after inoculation with Mix4 (‘Mix4’). In this case, a subset of 6 chicks was retained for the metabarcoding characterization of their gut microbiota. In the other two isolators, 35 chickens were reared until 27 days of age and infected with *Salmonella* at 7 days of age after inoculation of Mix4 (‘+Mix4+SE’) or not (‘+SE’). In this case, a subset of 14 chickens was retained for characterization of their gut microbiota by metabarcoding. (d), Relative abundance of the main OTU assigned to *Salmonella* sp. (representing >96% of the total abundance for this genus) in isolator (‘+Mix4+SE’) and 4 (‘+SE’) at each age category after infection (11, 14, 20 and 27 days of age, i.e. at 4, 7, 13 and 20 days post infection).

The Bray-Curtis β-diversity index comparison of taxonomic profiles revealed significant effects of both age and isolator (Permanova, *p* < 0.001 for both), reflecting the varied outcomes of GM development and breeding conditions across the four isolators. This analysis also emphasizes the substantial impact of *S*. Enteritidis and Mix4 inoculations on GM composition at every time point, regardless of infection status (comparison of the ‘+Mix4’ and ‘Ctrl’ isolators) (Figure 4(a-c)). Figure 4(b) shows that a single inoculation of four commensal bacteria had a short and long-term effect on the composition of the microbiota (*p* < 0.001 before and at 20 DoA and *p* = 0.034 at 27 DoA). Our analysis identified a total of 21, 15, 11, 20, 26, and 17 OTUs with significantly different abundances, at 4, 6, 11, 14, 20 and 27 DoA, respectively, between chickens that received Mix4 and those that did not; the most abundant of which are shown in Table 1. Notably, at 4 days of age, GM in the ‘+Mix4’ isolator showed increased levels of *Escherichia*, *Enterococcus*, and *Clostridium* sensu stricto, which may reflect the Mix4 composition itself, although in the absence of labeled strains, the metabarcoding cannot be used to confirm this. At 14 or 20 DoA, *Clostridium* sensu stricto and *Enterococcus* remained enriched in the ‘+Mix4’ isolator. Additionally, we observed enrichment for OTUs assigned to *Ruminococcus* torques group and *Blautia*, indicating either divergence between isolators or the long-term impact of Mix4 on GM composition.

**Table 1.** Main OTUs showing significant differential abundances between the controls and chickens inoculated with the Mix4 at one day of age.

		log2Fold Change*	padj	Family	Genus	Total abundance
4 days of age	Cluster_1	7.602083	9.64E–11	Enterobacteriaceae	*Escherichia-Shigella*	101403
	Cluster_4	7.014246	8.58E–06	Lactobacillaceae	*Pediococcus*	31423
	Cluster_6	8.603256	5.53E–11	Enterococcaceae	*Enterococcus*	31337
	Cluster_11	13.606643	3.09E–29	Lachnospiraceae	*unknown_genus*	28491
	Cluster_8	8.184793	1.13E–08	Lachnospiraceae	*[Ruminococcus]_torques_group*	26537
	Cluster_3	11.43788	4.17E–09	Clostridiaceae_1	*Clostridium_sensu_stricto_1*	24241
	Cluster_7	−4.69401	7.96E–03	Ruminococcaceae	*Flavonifractor*	22367
	Cluster_10	7.137203	5.78E–04	Lachnospiraceae	*Blautia*	19615
	Cluster_19	10.432867	8.40E–11	Lachnospiraceae	*[Ruminococcus]_torques_group*	10548
	Cluster_20	8.414882	8.14E–05	Clostridiaceae_1	*Clostridium_sensu_stricto_1*	7136
6 days of age	Cluster_7	−3.308705	1.23E–03	Ruminococcaceae	*Flavonifractor*	22367
	Cluster_10	−6.497225	5.35E–08	Lachnospiraceae	*Blautia*	19615
	Cluster_19	5.983594	2.99E–08	Lachnospiraceae	*[Ruminococcus]_torques_group*	10548
	Cluster_28	−8.361391	8.36E–13	Erysipelotrichaceae	*Erysipelatoclostridium*	7554
	Cluster_35	7.418431	3.59E–06	Lachnospiraceae	*Marvinbryantia*	6128
	Cluster_40	5.414976	4.29E–04	Ruminococcaceae	*DTU089*	4270
	Cluster_18	3.630338	9.35E–03	Clostridiaceae_1	*Clostridium_sensu_stricto_1*	3253
	Cluster_32	−4.286797	4.05E–03	Lachnospiraceae	*Sellimonas*	2783
	Cluster_43	4.951384	8.42E–03	Ruminococcaceae	*Ruminiclostridium_5*	1744
	Cluster_16	−3.607544	9.13E–03	Lachnospiraceae	*Anaerostipes*	1569
11 days of age	Cluster_24	−5.074642	5.14E–07	Lachnospiraceae	unknown_genus	9323
	Cluster_35	4.916488	3.75E–04	Lachnospiraceae	*Marvinbryantia*	6128
	Cluster_40	5.758536	3.72E–07	Ruminococcaceae	*DTU089*	4270
	Cluster_42	5.3012	2.63E–04	Lachnospiraceae	*Blautia*	3330
	Cluster_18	3.885406	3.81E–03	Clostridiaceae_1	*Clostridium_sensu_stricto_1*	3253
	Cluster_48	4.797527	3.75E–03	Lachnospiraceae	*Blautia*	3169
	Cluster_47	6.655841	6.19E–07	Ruminococcaceae	*Ruminiclostridium_9*	3128
	Cluster_32	5.098841	1.02E–06	Lachnospiraceae	*Sellimonas*	2783
	Cluster_43	4.674634	5.47E–03	Ruminococcaceae	*Ruminiclostridium_5*	1744
	Cluster_57	−4.507086	5.98E–04	Ruminococcaceae	unknown_genus	1713
14 days of age	Cluster_3	4.664522	6.64E–04	Clostridiaceae_1	*Clostridium_sensu_stricto_1*	24241
	Cluster_10	−3.946612	2.15E–03	Lachnospiraceae	*Blautia*	19615
	Cluster_12	−3.535008	1.72E–03	Lachnospiraceae	Multi-affiliation	17677
	Cluster_24	−3.032701	6.31E–03	Lachnospiraceae	unknown_genus	9323
	Cluster_28	−3.782455	2.80E–03	Erysipelotrichaceae	*Erysipelatoclostridium*	7554
	Cluster_35	4.699875	9.34E–04	Lachnospiraceae	*Marvinbryantia*	6128
	Cluster_36	−4.618438	5.27E–03	Lachnospiraceae	*[Ruminococcus]_torques_group*	5660
	Cluster_38	−4.264812	1.18E–03	Lachnospiraceae	unknown_genus	4244
	Cluster_42	6.211455	2.09E–05	Lachnospiraceae	*Blautia*	3330
	Cluster_41	−5.722887	6.64E–04	Lachnospiraceae	*Blautia*	3266
20 days of age	Cluster_6	3.117145	3.28E–04	Enterococcaceae	*Enterococcus*	31337
	Cluster_12	−6.393886	2.62E–10	Lachnospiraceae	Multi-affiliation	17677
	Cluster_19	3.930757	2.88E–04	Lachnospiraceae	[*Ruminococcus]_torques_group*	10548
	Cluster_24	−7.675636	4.57E–15	Lachnospiraceae	unknown_genus	9323
	Cluster_28	−6.661562	3.93E–07	Erysipelotrichaceae	*Erysipelatoclostridium*	7554
	Cluster_35	4.625334	6.12E–04	Lachnospiraceae	*Marvinbryantia*	6128
	Cluster_36	−6.723333	7.89E–06	Lachnospiraceae	*[Ruminococcus]_torques_group*	5660
	Cluster_38	−6.000532	5.04E–07	Lachnospiraceae	unknown_genus	4244
	Cluster_42	5.219572	3.70E–04	Lachnospiraceae	*Blautia*	3330
	Cluster_41	−5.780236	1.65E–05	Lachnospiraceae	*Blautia*	3266
27 days of age	Cluster_19	5.561029	6.29E–05	Lachnospiraceae	*[Ruminococcus]_torques_group*	10548
	Cluster_24	−6.252295	1.51E–06	Lachnospiraceae	unknown_genus	9323
	Cluster_28	−6.023934	1.10E–04	Erysipelotrichaceae	*Erysipelatoclostridium*	7554
	Cluster_36	−5.733201	3.35E–03	Lachnospiraceae	*[Ruminococcus]_torques_group*	5660
	Cluster_38	−6.362761	2.37E–05	Lachnospiraceae	unknown_genus	4244
	Cluster_42	5.583232	7.75E–04	Lachnospiraceae	*Blautia*	3330
	Cluster_41	−5.684589	9.59E–04	Lachnospiraceae	*Blautia*	3266
	Cluster_48	5.883437	7.90E–03	Lachnospiraceae	*Blautia*	3169
	Cluster_47	4.942368	4.19E–03	Ruminococcaceae	*Ruminiclostridium_9*	3128
	Cluster_32	5.035097	1.22E–04	Lachnospiraceae	*Sellimonas*	2783

*Positive log2 fold changes correspond to the OTUs enriched in isolator Mix4. We only report here the 10 main OTUs, determined by their total abundance across the time series.

**Figure 5.**
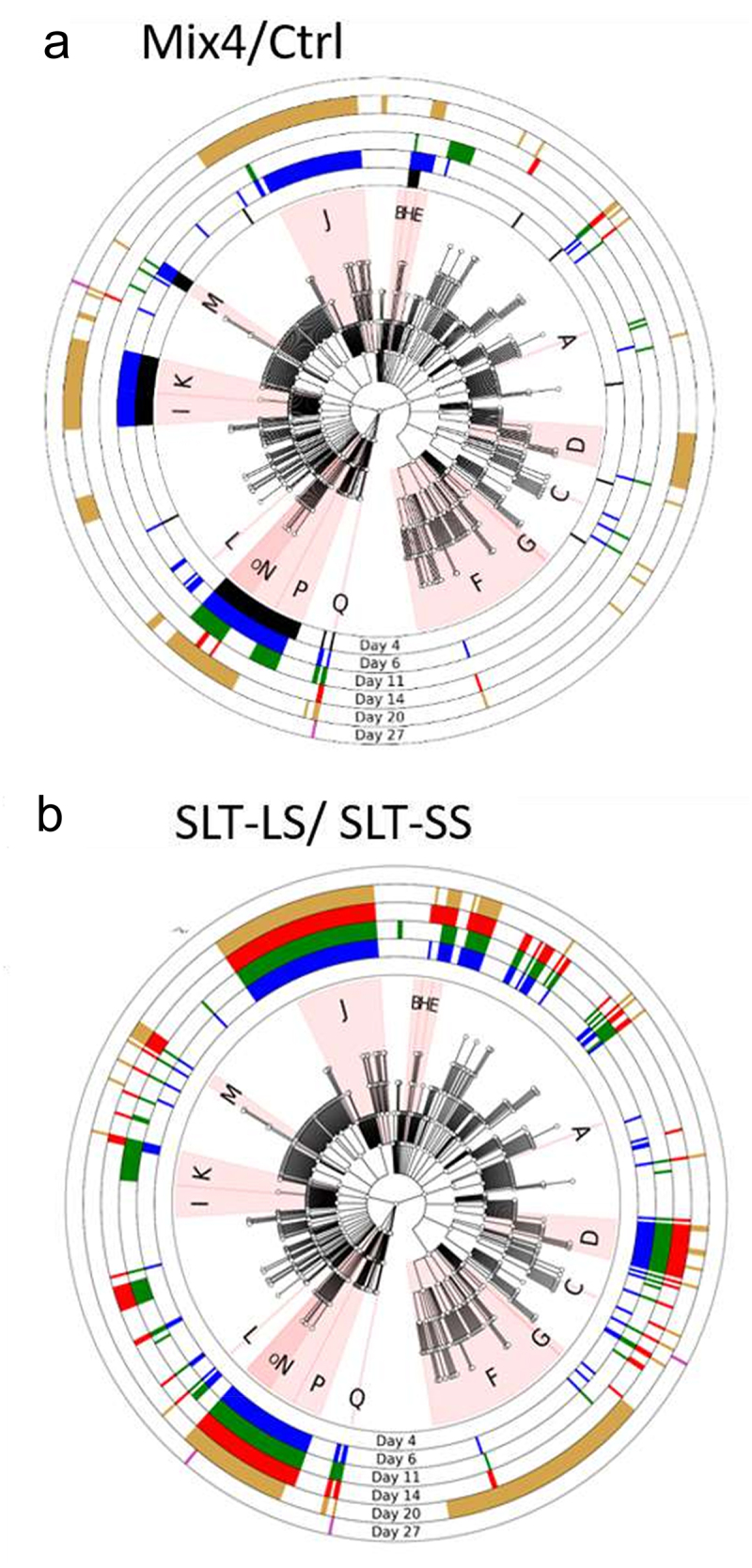
Cladogram presenting a hierarchical overview of the MetaCyc pathways detected after the Mix4 treatment (a) and by comparison of the SLT-LS and SLT-SS (b). the metabolic pathways, corresponding to the MetaCyt curated database, were detected using PICRUSt2 after metabarcoding analysis. Central nodes represent general pathways while the progressively peripheral nodes represent more specific pathways. The outer rings indicate the presence/absence of this pathway at different time-points. The colors, which varied depending of the days of age of chickens, represent differentially expressed pathways of particular interest. The letters correspond respectively to the following nodes : A; L-ornithine biosynthesis; B; carboxylate biosynthesis; C; ubiquinol biosynthesis; D; fatty acids biosynthesis; E; amide, amidine, amine and polyamine biosynthesis; F; secondary metabolite biosynthesis; G; siderophore and metallophore biosynthesis; H; tetrapyrrole biosynthesis; I; amide, amidine, amine and polyamine degradation; J; aromatic compounds degradation; K; carboxylic acid degradation; L; fatty acids degradation; M; nitrogen compounds metabolism; N; generation of precursor metabolites and energy; O; fermentation; P; glycan pathways; Q; superpathways.

To assess whether the changes in taxa affected the functional roles of the microbiota, we analyzed the data using PICRUSt2.^26^ Figure 5, presented as a cladogram, illustrates all metabolic pathways found in the MetaCyc curated database (at the date of February 13th, 2024) which includes experimentally defined metabolic pathways from all domains of life. Figure 5(a) shows numerous metabolic pathways with significantly different abundances between the ‘+Mix4’ and the ‘Ctrl’ groups from 4 days of age (38 pathways in the black circle) and up to 20 (76 pathways in orange circle) and 27 days of age (2 pathways in purple circle). It is interesting to note that the greatest number of modified metabolic pathways (112 pathways in the blue circle) was obtained at 6 days of age, which is just before infection, for the infected groups. These pathways correspond to specific MetaCyc categories, as indicated by letters in Figure 5 (J: aromatic compounds degradation, P: carbohydrate biosynthesis pathways, D, L, O: fermentation and short chain fatty acids (SCFA) production, Q: anaerobic respiration pathways). A detailed analysis showed that Mix4 inoculation promoted the ‘degradation of aromatic compounds’ pathways at 6 days of age, while pathways related to ‘SCFA production’ and ‘fermentation’ were more prevalent in the Ctrl group at 4 and 6 days of age. Additionally, pathways related to anaerobic respiration such as “menaquinol and ubiquinol biosynthesis” were more prominent in the ‘Ctrl’ group at 6 days of age. These results suggest that the Mix4 modifies not only the GM composition but also the associated metabolic pathways, both in the short and long term.

When we compare the GM compositions between the ‘Ctrl’ and ‘+SE’ isolators, we found significant differences in β-diversities at every time point after infection (*p* < 0.001) (Figure 4(a)). Table 2 highlights the main OTUs with differential abundances, among the 32, 34, 41, and 17 OTUs detected at 11, 14, 20 and 27 days of age, respectively. As expected, *Salmonella* was among the differentially abundant OTUs at all time-points. Among the other Enterobacteriaceae, we can observe, just after infection (4 Dpi or 11 DoA), a lower abundance of *Escherichia*, in the ‘+SE’ group, compared to the ‘Ctrl’ group. This trend later reversed, with a higher abundance of another Enterobacteriaceae (*Klebsiella*) in the infected group. Additionally, genera such as *Butyricoccus*, *Oscillibacter*, and *Anaerostipes* were constantly enriched in the ‘+SE’ isolator, whereas several genera from the Lachnospiraceae family were enriched in the ‘Ctrl’ isolator. However, as we observed certain differences between the two groups at 4 and 6 days of age, it is difficult to determine whether these differences are linked to infection by *S*. Enteritidis or to the fact that the differences observed after infection are due to the small difference present before infection.

**Table 2.** Main OTUs showing significant differential abundances between the control chickens and chickens inoculated with *Salmonella* at 7 days of age.

		log2 Fold Change*	padj	Family	Genus	Total abundance
11 days of age	Cluster_1	11.482552	7.574324e-22	Enterobacteriaceae	*Escherichia-Shigella*	86221
	Cluster_8	7.079783	1.950891e-19	Lachnospiraceae	*[Ruminococcus] torques group*	32666
	Cluster_11	5.988923	4.890426e-12	Lachnospiraceae	unknown genus	29291
	Cluster_10	13.115918	1.439677e-18	Lachnospiraceae	*Blautia*	22279
	Cluster_7	9.295750	6.006828e-22	Ruminococcaceae	*Flavonifractor*	21521
	Cluster_17	−8.996010	3.595334e-05	Ruminococcaceae	*Butyricicoccus*	14886
	Cluster_27	−14.522571	2.816261e-10	Ruminococcaceae	*Oscillibacter*	11314
	Cluster_4	7.497209	5.862687e-07	Lactobacillaceae	*Pediococcus*	9627
	Cluster_24	18.825851	1.206431e-31	Lachnospiraceae	unknown genus	9598
	Cluster_26	−6.211751	1.197498e-05	Enterobacteriaceae	*Salmonella*	6242
14 days of age	Cluster_8	−1.628803	1.166537e-03	Lachnospiraceae	*[Ruminococcus] torques group*	32666
	Cluster_2	−10.814914	4.094032e-22	Enterobacteriaceae	*Klebsiella*	23500
	*Cluster_6*	−2.700800	4.467592e-03	Enterococcaceae	*Enterococcus*	19568
	Cluster_12	7.813864	4.419502e-22	Lachnospiraceae	Multi-affiliation	18666
	Cluster_17	−4.820679	3.566471e-06	Ruminococcaceae	*Butyricicoccus*	14886
	Cluster_14	−7.983574	2.712594e-16	Enterobacteriaceae	*Klebsiella*	12801
	Cluster_27	−8.695676	8.214947e-13	Ruminococcaceae	*Oscillibacter*	11314
	Cluster_24	6.500381	4.923764e-12	Lachnospiraceae	unknown genus	9598
	Cluster_16	−5.064924	2.263503e-08	Lachnospiraceae	*Anaerostipes*	6706
	Cluster_26	−7.809743	2.588012e-15	Enterobacteriaceae	*Salmonella*	6242
20 days of age	Cluster_2	−7.097424	1.58E–14	Enterobacteriaceae	*Klebsiella*	23500
	Cluster_12	8.745618	1.13E–27	Lachnospiraceae	Multi-affiliation	18666
	Cluster_17	−5.301376	2.83E–07	Ruminococcaceae	*Butyricicoccus*	14886
	Cluster_14	−5.913102	1.19E–09	Enterobacteriaceae	*Klebsiella*	12801
	Cluster_27	−6.266165	1.19E–09	Ruminococcaceae	*Oscillibacter*	11314
	Cluster_24	8.409352	2.32E–20	Lachnospiraceae	unknown	9598
	Cluster_16	−3.228757	3.28E–04	Lachnospiraceae	*Anaerostipes*	6706
	Cluster_26	−6.176414	1.10E–19	Enterobacteriaceae	*Salmonella*	6242
	Cluster_36	9.482414	9.49E–16	Lachnospiraceae	*[Ruminococcus] torques group*	5104
	Cluster_34	−6.526223	2.32E–08	Ruminococcaceae	Multi-affiliation	4229
27 days of age	Cluster_2	−7.342308	3.357631e-11	Enterobacteriaceae	*Klebsiella*	23500
	Cluster_14	−7.417478	1.181367e-09	Enterobacteriaceae	*Klebsiella*	12801
	Cluster_27	−6.035410	3.545815e-08	Ruminococcaceae	*Oscillibacter*	11314
	Cluster_16	−2.575678	6.648040e-03	Lachnospiraceae	*Anaerostipes*	6706
	Cluster_26	−6.562604	3.634769e-14	Enterobacteriaceae	*Salmonella*	6242
	Cluster_19	−6.405544	1.900248e-07	Lachnospiraceae	*[Ruminococcus] torques group*	6186
	Cluster_36	7.155219	1.320247e-09	Lachnospiraceae	*[Ruminococcus] torques group*	5104
	Cluster_34	−3.766911	4.408238e-03	Ruminococcaceae	Multi-affiliation	4229
	Cluster_38	8.932455	3.187658e-14	Lachnospiraceae	unknown genus	4182
	Cluster_15	−3.843210	1.005126e-03	Lachnospiraceae	*Lachnospiraceae FE2018 group*	3682

*Positive log2 fold changes correspond to the OTUs enriched in isolator ‘Ctrl’. We only report here the 10 main OTUs determined by their total abundance across the time series. padj= adjusted *p* value.

Similar to the effects of Mix4 inoculation, infection with *S*. Enteritidis led to significant differences in numerous metabolic pathways between the ‘Ctrl’ and ‘+SE’ groups at each time point after infection (218, 87, 149, and 13 pathways at 11, 14, 20, and 27 days of age, respectively). Overall, this result shows the dramatic impact of infection on the composition of the microbiota and the metabolic functions it supports especially in the first 2 weeks after infection, although we cannot rule out the possibility that this could be related to an ‘isolator effect’ (i.e. isolator-related differences). This has been taken into account later in the study.

When we investigated the impact of the Mix4 inoculation on *S*. Enteritidis infection, we observed, Figure 4(c), significant differences in the β-diversity indexes at each age. Before infection, the main OTUs showing differential abundances between the ‘+Mix4+SE’ and ‘+SE’ isolators were assigned to the genera *Escherichia*, *Klebsiella*, and *Clostridium* sensu stricto (Table 3). Notably, the most differentially abundant OTUs are anaerobic bacteria known to produce short-chain fatty acids (SCFA), key metabolic regulators of gut health and homeostasis. After infection, *Faecalibacterium* and *Lachnospiraceae* FE2018 group were more abundant in the ‘+Mix4+SE’group, while *Butyricoccus*, another SCFA producer, was more abundant in the ‘+SE’group, at 6 days of age.

**Table 3.** Main OTUs showing significant differential abundances between the chickens infected with *Salmonella* at 7 days of age after or not an initial inoculation of the Mix4 at one day of age.

		log2 Fold Change*	padj	Family	Genus	Total abundance
4 days of age	Cluster_1	12.584901	2.64E–33	Enterobacteriaceae	*Escherichia-Shigella*	48793
	Cluster_2	−6.176252	8.51E–03	Enterobacteriaceae	*Klebsiella*	45255
	Cluster_14	−7.651239	4.19E–04	Enterobacteriaceae	*Klebsiella*	24579
	Cluster_9	7.718807	6.60E–09	Clostridiaceae_1	*Clostridium_sensu_stricto_1*	21680
	Cluster_18	10.651333	8.92E–06	Clostridiaceae_1	*Clostridium_sensu_stricto_1*	12535
	Cluster_13	11.883421	1.48E–13	Clostridiaceae_1	*Clostridium_sensu_stricto_1*	11345
	Cluster_11	−8.117112	3.31E–05	Lachnospiraceae	unknown_genus	7469
	Cluster_20	6.011752	4.82E–05	Clostridiaceae_1	*Clostridium_sensu_stricto_1*	7410
	Cluster_33	4.998913	2.42E–03	Clostridiaceae_1	*Clostridium_sensu_stricto_1*	6828
	Cluster_3	7.033638	6.82E–09	Clostridiaceae_1	*Clostridium_sensu_stricto_1*	6306
6 days of age	Cluster_1	1.694578	2.32E–03	Enterobacteriaceae	*Escherichia-Shigella*	48793
	Cluster_2	−3.469507	1.04E–03	Enterobacteriaceae	*Klebsiella*	45255
	Cluster_14	−3.58289	4.24E–04	Enterobacteriaceae	*Klebsiella*	24579
	Cluster_9	−1.939517	3.95E–03	Clostridiaceae_1	*Clostridium_sensu_stricto_1*	21680
	Cluster_13	3.826454	1.88E–05	Clostridiaceae_1	*Clostridium_sensu_stricto_1*	11345
	Cluster_11	−2.76462	3.50E–03	Lachnospiraceae	unknown_genus	7469
	Cluster_33	−2.142787	5.21E–03	Clostridiaceae_1	*Clostridium_sensu_stricto_1*	6828
	Cluster_22	−3.165972	9.73E–06	Peptostreptococcaceae	*Clostridioides*	4083
	Cluster_17	−7.300275	1.91E–05	Ruminococcaceae	*Butyricicoccus*	2266
	Cluster_25	4.039372	9.70E–05	Ruminococcaceae	*Ruminococcus_1*	2239
11 days of age	Cluster_1	12.399227	1.92E–25	Enterobacteriaceae	*Escherichia-Shigella*	101749
	Cluster_5	21.155336	1.85E–41	Ruminococcaceae	*Faecalibacterium*	44664
	Cluster_4	20.766941	1.84E–35	Lactobacillaceae	*Pediococcus*	42572
	Cluster_11	7.653684	2.46E–64	Lachnospiraceae	unknown_genus	30398
	Cluster_8	4.784897	1.60E–11	Lachnospiraceae	*[Ruminococcus]_torques_group*	29241
	Cluster_3	15.743623	5.74E–16	Clostridiaceae_1	*Clostridium_sensu_stricto_1*	26691
	Cluster_6	13.327381	2.03E–20	Enterococcaceae	*Enterococcus*	26283
	Cluster_7	7.571996	1.10E–36	Ruminococcaceae	*Flavonifractor*	18757
	Cluster_15	9.492674	3.35E–11	Lachnospiraceae	*Lachnospiraceae_FE2018_group*	18481
	Cluster_17	−4.792356	3.83E–04	Ruminococcaceae	*Butyricicoccus*	14724
14 days of age	Cluster_5	9.236231	3.18E–19	Ruminococcaceae	*Faecalibacterium*	44664
	Cluster_4	5.034865	1.29E–05	Lactobacillaceae	*Pediococcus*	42572
	Cluster_8	−1.48055	2.90E–03	Lachnospiraceae	*[Ruminococcus]_torques_group*	29241
	Cluster_2	−7.265631	9.72E–17	Enterobacteriaceae	*Klebsiella*	23572
	Cluster_15	10.582063	1.43E–33	Lachnospiraceae	*Lachnospiraceae_FE2018_group*	18481
	Cluster_17	−7.883887	4.04E–19	Ruminococcaceae	*Butyricicoccus*	14724
	Cluster_10	2.339149	3.66E–03	Lachnospiraceae	*Blautia*	13010
	Cluster_14	−6.7548	9.00E–15	Enterobacteriaceae	*Klebsiella*	12832
	Cluster_16	2.550652	1.38E–04	Lachnospiraceae	*Anaerostipes*	11553
	Cluster_27	−7.697138	1.59E–16	Ruminococcaceae	*Oscillibacter*	11362
20 days of age	Cluster_1	5.724529	2.68E–12	Enterobacteriaceae	*Escherichia-Shigella*	101749
	Cluster_5	11.006636	6.34E–24	Ruminococcaceae	*Faecalibacterium*	44664
	Cluster_4	5.526813	3.36E–06	Lactobacillaceae	*Pediococcus*	42572
	Cluster_11	−1.057028	2.15E–03	Lachnospiraceae	unknown_genus	30398
	Cluster_3	4.039704	4.80E–03	Clostridiaceae_1	*Clostridium_sensu_stricto_1*	26691
	Cluster_6	5.11562	4.21E–07	Enterococcaceae	*Enterococcus*	26283
	Cluster_2	−5.141617	7.50E–08	Enterobacteriaceae	*Klebsiella*	23572
	Cluster_15	8.915778	1.67E–24	Lachnospiraceae	*Lachnospiraceae_FE2018_group*	18481
	Cluster_17	−4.674498	1.14E–08	Ruminococcaceae	*Butyricicoccus*	14724
	Cluster_14	−5.100881	2.24E–07	Enterobacteriaceae	*Klebsiella*	12832
27 days of age	Cluster_5	7.709119	4.43E–12	Ruminococcaceae	*Faecalibacterium*	44664
	Cluster_6	2.877993	8.22E–03	Enterococcaceae	*Enterococcus*	26283
	Cluster_2	−3.137906	1.20E–03	Enterobacteriaceae	*Klebsiella*	23572
	Cluster_17	−3.51456	4.41E–05	Ruminococcaceae	*Butyricicoccus*	14724
	Cluster_14	−3.065228	1.48E–03	Enterobacteriaceae	*Klebsiella*	12832
	Cluster_27	−4.789005	8.65E–08	Ruminococcaceae	*Oscillibacter*	11362
	Cluster_25	6.425881	2.48E–11	Ruminococcaceae	*Ruminococcus_1*	7923
	Cluster_30	7.560867	4.58E–09	Clostridiales_vadinBB60 _group	unknown_genus	6287
	Cluster_44	6.345545	3.45E–08	Lachnospiraceae	*GCA-900066575*	3635
	Cluster_22	3.290602	2.90E–06	Peptostreptococcaceae	*Clostridioides*	3399

*Positive log2 fold changes correspond to the OTUs enriched in infected chicks which received Mix4. We only report here the 10 main OTUs, determined by their total abundance across the time series.

padj= adjusted p value

These differences in OTUs suggest distinct metabolic pathways in the gut microbiota of the two groups, both before and after infection, potentially explaining the varying levels of *Salmonella* colonization. This was supported by MetaCyc pathway analysis, which revealed 62, 183, 203, 138, 154, 66 different pathways at 4, 6, 11, 14, 20, 27 days of age, respectively, between the ‘+Mix4+SE’ and ‘+SE’ groups. These numbers of pathways are close to those obtained between infected and uninfected chicks. This suggests that Mix4 can induce long-term changes in OTUs and metabolic functions, even during *S*. Enteritidis infection.

The observed differences in OTU abundances and metabolic pathways may be attributed to several factors: (1) the presence of various environmental bacteria in the non-sterile isolators at the beginning of the experiment, particularly affecting OTUs assigned to *Escherichia* and *Klebsiella* and responsible of a putative ‘isolator effect’; (2) the influences of Mix4 inoculation on GM β-diversity; and (3) the effect of *Salmonella* infection in both Mix4-inoculated and non-inoculated chickens. Supporting the third factor, we found a significant drop in *Salmonella* relative abundances, as measured by metabarcoding, in the ‘+Mix4+SE’ isolator compared to the ‘+SE’ isolator, at 20 (*p* < 0.001) and 27 days of age (*p* = 0.007) (Figure 4(d)). Under a mixed linear model framework, analyzing the abundance of the predominant *Salmonella* OTU (which represents 96% of the total abundance for this genus) over the entire time series, we observed a significant impact of Mix4 inoculation on *Salmonella* OTU abundance (*p* = 0.015), alongside the effects of *Salmonella* inoculation, age of the chicken, and individual variability.

In summary, the differences in OTU abundances and metabolic pathways between the different groups could be linked to Mix4 inoculation and *Salmonella* infection, although we cannot exclude a role for the batch effect linked to the independent development of microbiota in the different isolators, during the first days of age.

### *Impact of Mix4 inoculation on the short and long term* Salmonella *Excretion*

**Figure 6.**
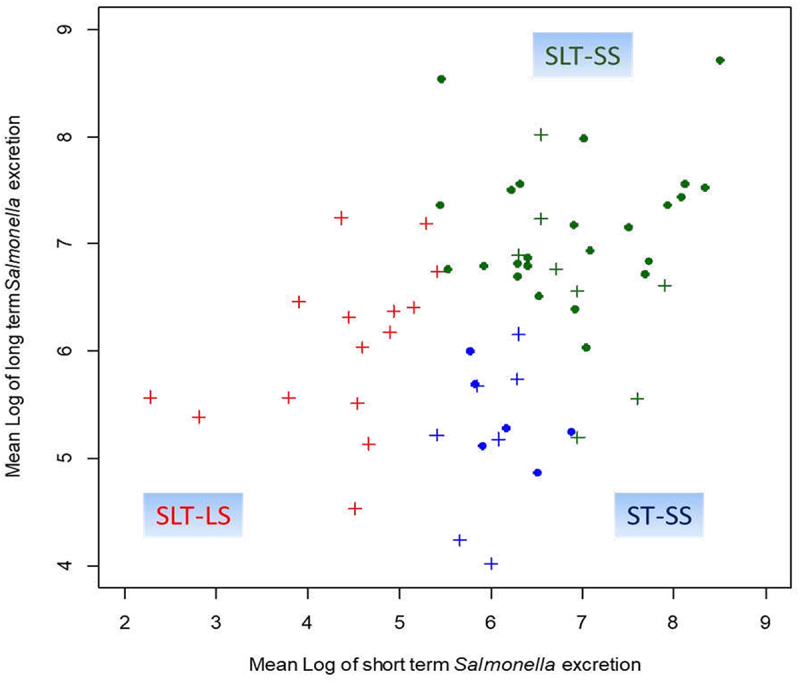
Biplot of the *Salmonella* excretion by chicks. Biplot represents the short term (days 4 and 7 post infection) and long term (days 13 and 20 post infection) *Salmonella* excretion of the chicks reared in isolator ‘+Mix4+SE’ (crosses) and ‘+SE’ (dots). A ward classification made on the basis of short and long term excretion levels lead to a 3-classes distribution: the short and long term super shedders (SLT-SS, in green), the short and long term low shedders (SLT-LS, in red) and the short term super shedders (ST-SS, in blue).

As we have recently demonstrated in pigs^17^, the LS and SS phenotypes are mainly influenced by early interactions between immune response and GM composition just after infection, it was decided in the present analysis to classify animals according to their level of *Salmonella* colonization at early (11 and 14 DoA) and late (20 and 27 DoA) stages of infection.^17^ Moreover, to assess the effect of Mix4 on *Salmonella* colonization, we combined the two infection conditions before performing the hierarchical clustering. This analysis enabled us to classify chicks into three groups based on their *Salmonella* shedding patterns (Figure 6). The first group, labeled as Short- and Long-Term Low Shedders (SLT-LS), consisted of chicks that constantly exhibited low levels of *Salmonella* shedding both in the short and long term. Notably, the SLT-LS group only included chicks that received the Mix4 inoculation. The second group, labeled as Short-Term Super Shedders (ST-SS), included chicks with high levels of *Salmonella* excretion that either remained stable or decreased over time. This group had a comparable number of chicks with or without Mix4 inoculation. The third group, labeled as Short- and Long-Term Super Shedders (SLT-SS), comprised chicks exhibiting high initial *Salmonella* shedding levels that increased over time. This group included eight chicks that received Mix4 and 24 that were infected without Mix4. These three categories correspond to contrasting patterns of excretion over time (Supplementary Figure S2). These findings underscore the protective effect of Mix4 against *Salmonella* colonization, leading to a higher proportion of SLT-LS. This may be related to its influence on the GM composition and/or function, which was then further examined.

### The GM composition is correlated to the shedding phenotype of animals

Many OTUs were significantly different between these three groups of excretion (i.e. the SLT-LS, SLT-SS, ST-SS). For example, Supplementary Table SI shows the main OTUs showing significant differences in abundance between SLT-SS and SLT-LS chickens after infection. However, since our previous findings indicate that GM composition prior to infection determines susceptibility to *Salmonella* colonization^18^, we aimed to identify the commensal bacteria involved in *Salmonella* implantation and the barrier effect. To achieve this, we analyzed the GM composition both before infection and between the two extreme groups: Short- and Long-Term Low Shedders (SLT-LS) and Short- and Long-Term Super Shedders (SLT-SS). Twenty OTUs were differentially abundant at 6 days of age, whereas we did not observe differences at 4 days of age (Table 4). Although no bacterial genus was enriched in the SLT-LS group, many families were enriched in the chicks that will become the most susceptible animals to *Salmonella* (SLT-SS). Among them we observed strict anaerobic bacteria (e.g. Lachnospiraceae, Peptostreptococcaceae, Ruminococceae) known to produce SCFA and facultative anaerobes (e.g. Enterobacteriaceae, Erysipelotrichaceae). Interestingly, Erysipelotrichaceae have been associated with host metabolic disorders and inflammatory diseases.^27^

**Table 4.** Main OTUs showing significant differential abundances between the short- and long-term super shedders (SLT-SS) and the short- and long-term low shedders (SLT-LS) before infection.

		log2 Fold Change*	padj	Family	Genus
4 days of age	*No differences*				
6 days of age	Cluster_2	−11.17	5.05×10^−25^	Enterobacteriaceae	*Klebsiella*
	Cluster_14	−9.74	1.48×10^−20^	Enterobacteriaceae	*Klebsiella*
	Cluster_83	−8.18	1.47×10^−10^	Enterobacteriaceae	*Escherichia-Shigella*
	Cluster_138	−7.65	4.27×10^−10^	Enterobacteriaceae	*Escherichia-Shigella*
	Cluster_55	−7.95	7.78×10^−10^	Lachnospiraceae	*Tyzzerella3*
	Cluster_51	−6.71	1.71×10^−8^	Lachnospiraceae	*CHKCI001*
	Cluster_77	−10.51	4.01×10^−8^	Peptostreptococcaceae	*Paeniclostridium*
	Cluster_26	−6.75	1.52×10^−7^	Enterobacteriaceae	*Salmonella*
	Cluster_46	−9.89	3.44×10^−7^	Erysipelotrichaceae	*Erysipelatoclostridium*
	Cluster_544	−6.42	2.21×10^−6^	Enterobacteriaceae	*Escherichia-Shigella*
	Cluster_436	−5.48	2.33×10^−5^	Enterobacteriaceae	*Klebsiella*
	Cluster_602	−6.64	1.61×10^−4^	Lachnospiraceae	*CHKCI001*
	Cluster_17	−6.80	2.05×10^−4^	Ruminococcaceae	*Butyricicoccus*
	Cluster_129	−5.96	7.42×10^−4^	Clostridiaceae 1	*Clostridium sensu stricto 1*
	Cluster_677	−6.39	8.63×10^−4^	Lachnospiraceae	*Epulopiscium*
	Cluster_336	−5.82	8.63×10^−4^	Enterococcaceae	*Enterococcus*
	Cluster_290	−4.18	9.38×10^−4^	Lactobacillaceae	*Pediococcus*
	Cluster_953	−4.30	2.01×10^−3^	Lachnospiraceae	*[Ruminococcus] torques group*
	Cluster_13	2.65	7.18×10^−3^	Clostridiaceae 1	*Clostridium sensu stricto 1*
	Cluster_35	−4.95	8.34×10^−3^	Lachnospiraceae	*Marvinbryantia*

*Positive log2 fold changes correspond to the OTUs enriched in the SLT-LS chickens.

padj = adjusted p value

**Figure 7.**
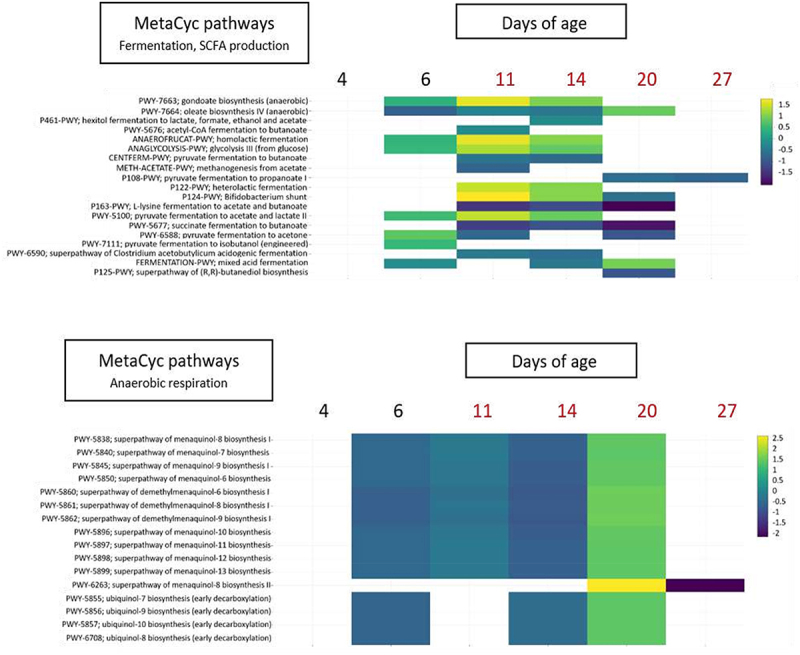
Heatmaps representing the most relevant differentially expressed metabolic pathways between the SLT-SS and SLT-LS at different time-points. the metabolic pathways, corresponding to the MetaCyt curated database, were detected using PICRUSt2 after metabarcoding analysis. The heatmap focus on two particular and significant metabolic pathways corresponding to “fermentation and SCFA production” and to anaerobic respiration”. Each line corresponds to a more specific metabolic pathway of the general metabolic pathway. Each column corresponds to the GM analysis at a specific time-point. The positive log Fold changes correspond to the pathways enriched in the short and long term super shedders (SLT-SS) compared to the short and long term low shedders (SLT-LS).

If we compared the GM metabolic pathways found in the SLT-LS and in the SLT-SS groups, we observed a massive difference before and after *Salmonella* infection with 0, 226, 222, 211, 114 and 5 metabolic pathways differentially expressed at 4, 6, 11, 14, 20 and 27 days of age, respectively. This result reveals that just before and just after infection the functions present in the GM were very different between chicks that are susceptible (SLT-SS) or resistant (SLT-LS) to *Salmonella* colonization (Figure 5(b)). These differences are related to numerous pathways related to fermentation, short-chain fatty acid (SCFA) production, degradation of aromatic compounds, anaerobic respiration, and glycan metabolism (Figure 5(c)). Such dramatic variations in the GM’s functional capabilities could play a crucial role in *Salmonella’s* ability to overcome barrier effect. This is particularly evident in the metabolic pathways associated with anaerobic respiration, as illustrated in Figure 7. We can observe that virtually all metabolic pathways involved in menaquinol (vitamin K2) biosynthesis are increased in the SLT-SS, in contrast to chicks belonging to the SLT-LS group. This is also observed after infection (11 and 14 DoA). Similarly, another metabolic pathways involved in anaerobic respiration and even aerobic respiration: the ubiquinol (coenzyme Q10) biosynthesis pathway, is also more highly expressed before infection in chicks belonging to the SLT-SS group compared with the SLT-LS group. Interestingly, both the metabolic pathways for menaquinol (vitamin K2) and ubiquinol (coenzyme Q10) biosynthesis play crucial roles in the energy metabolism of intestinal bacteria and especially in the electron transport chain.^28^ However, later (20 DoA) the trend is reversed as these pathways related to anaerobic respiration are more expressed in chicks belonging to the SLT-LS group compared to the group SLT-SS. These differences could be related to a modification of the immune response and therefore play an important role in *Salmonella* implantation in the intestine. As for the metabolic pathways linked to the production of SCFA, the picture is less clear before infection, since although we have several differences between the two groups, they do not go in the same direction and are less strong than those linked to anaerobic respiration. This is even more variable after infection, which prevents us from concluding on a potential role for these metabolic pathways in susceptibility to *Salmonella* colonization.

**Figure 8.**
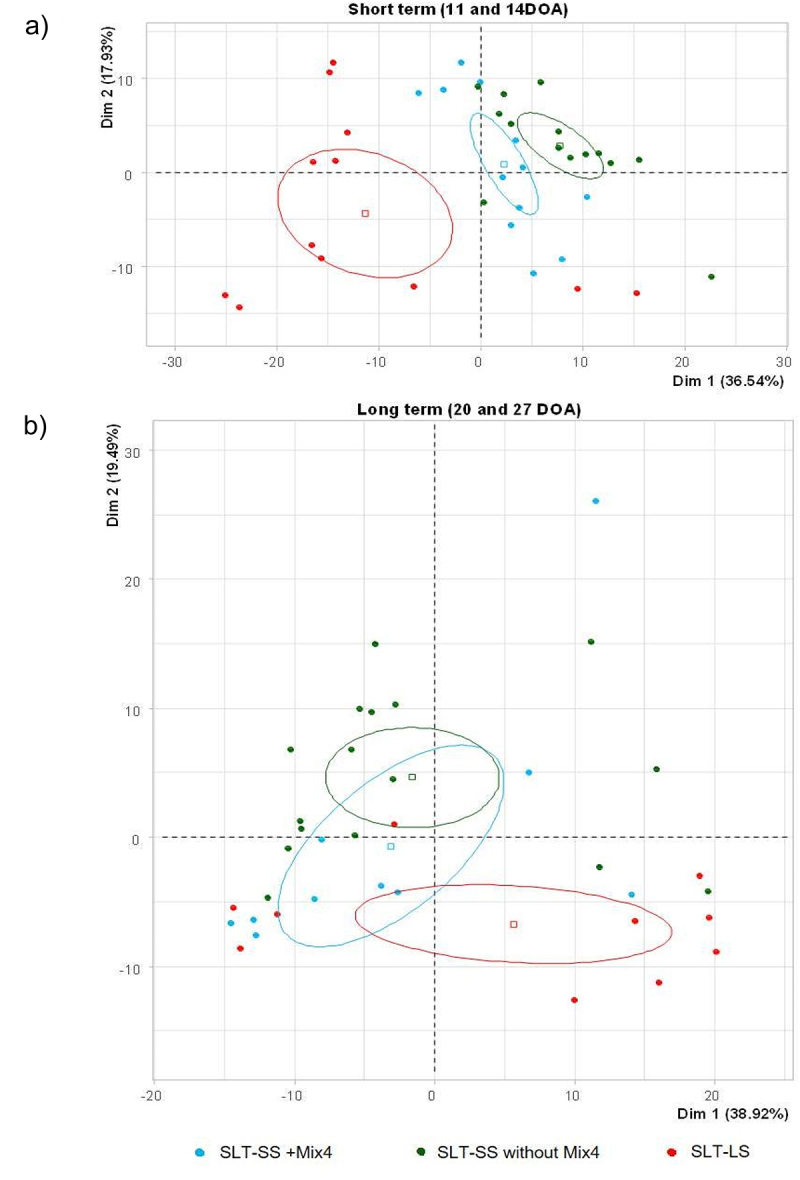
PCA summarizing functional compositions of the gut microbiota in SLT-SS (with or without Mix4) and SLT-LS chickens, at short term (a) and long term (b) (i.e. timepoints 11 and 13 DOA for the short term and 20 and 27 DOA for the long term). Functions were inferred on the basis of taxonomy using PICRUSt2.^26^

A particular focus was paid to animals that received Mix4 but, despite this, exhibited high levels of *Salmonella* excretion, i.e., those belonging to the SLT-SS category (8 out of 32 individuals). Interestingly, in the PCA, the GM functions of these chickens were closer to those of other SLT-SS chickens than to SLT-LS (Figure 8). This is particularly observed just before and after infection, although later on the functions carried by SLT-SS animals that received Mix4 are different from those of animals that received Mix4 but are SLT-LS. This similarity between SLT-SS chickens with and without Mix4 was less observed at the taxonomical level (not shown), illustrating the interest of functional inference in avoiding functional redundancy between taxa. For example, in the short term, we observed that pathways related to anaerobic respiration (i.e., menaquinol and ubiquinol biosynthesis pathways) were highly correlated with principal component 1 (R₀ > 0.85), whose positive values were also associated with the SLT-SS phenotype, regardless of the treatment received by the individuals (mean value: 7.78, v-test = 3.19 and mean value: 2.29, v-test = 0.83 for SLT-SS chickens with and without Mix4 respectively). A reverse pattern was observed in the long term (Figure 8), where anaerobic respiratory pathways were instead associated with principal component 1 (R₀ > 0.85) and the SLT-LS category (mean value: 5.64; v-test = 1.77) rather than with the SLT-SS category (mean value: –3.11, v-test = −0.98 and mean value: −1.56, v-test = −0.72 for SLT-SS chickens with and without Mix4 respectively). Once again, this supports the hypothesis that the shedding categories were primarily driven by the functional composition of the GM. Mix4 modulates the GM, leading mainly to the SLT-LS phenotype but sometimes to the SLT-SS phenotype.

Surprisingly, few differences in differential metabolic pathways of the GM were observed at 4 DoA, just after Mix4 inoculation, and the most significant differences were observed just before infection (at 6 DoA), suggesting that it is not Mix4 per se that is responsible for these metabolic differences. Consequently, the host defenses themselves need to be investigated. Indeed, the presence of super-shedders observed in both Mix4-inoculated and non-inoculated groups (Figure 6) suggests that factors other than Mix4 may also influence *Salmonella* colonization levels, such as the immune response or status.

### The Mix4 inoculation has an impact on the immune response

We have shown that modulation of the GM composition and function influences *Salmonella* colonization and excretion. This effect could be direct or via modulation of the immune response. To determine whether the Mix4 inoculation modulates the immune response, the gene expression kinetics of 74 immune-related genes was analyzed by RT-qPCR, from blood samples collected at various time points (Table 5). These genes had been chosen to analyze both the innate and adaptive immune response (Supplementary Table SII). A total of 243 individuals across all timepoint could be characterized in this way, for 66 immune genes.

**Table 5.** Relative gene expression in blood of chicks that received or not the Mix4.

Gene	FC at 4DoA	FC at 6DoA	FC at 11 DoA	FC at 14 DoA	FC at 20 DoA	FC at 27 DoA
*ALOX5AP*		2,1				
*CCR2*		−2,4				
*CD28*		−2,0				
*CD69*		2,6				
*CD86*						−2.8
*CEBPB*		−2,3				
*CISH*			−4,8			
*CSF2RA*		−2,0				
*GAL6*						2.2
*HMOX1*		2,1				
*IRF7*		4,1				
*SOCS3*			−2.9			
*STAT6*		−2,2				
*IL15*		2.2	2,8	2,5	2.0	
*MDA5*	2.1		2,0	2,0		
*MX1*	12.1	6.6	12,1	11,7	8.7	
*CD180*					−2.4	−3.1
*SOCS1*		3.1		2.7		
*TNFRSF1A*		−2.5	−2.1			

*FC represents the ratio between non-infected chicks treated or not with Mix4. Positive FC means that the gene is over-expressed in the ‘+Mix4’ group. Ratio > 2.0, with a *p* value < 0.05 were considered.

Three days after administration of four commensal bacteria (4 DoA), significant differences in the expression of *MX1* and *MDA5* (*IFIH1*) were observed, indicating the induction of a strong type I interferon signature in Mix4-inoculated chicks compared to controls. By 5 days after Mix4 inoculation (6 DoA), many immune genes were differentially expressed, peaking just before *Salmonella* infection at 7 DoA. This timing may explain the different outcomes of *Salmonella* colonization via a coherent picture of these gene expressions. MDA5 and IRF7, which are known to be induced by bacterial challenge, indeed activate type I interferon, which in turn activates M×1 and CD69.^29^ This immune response may be related to direct effects of Mix4 or to changes in microbiota composition. In addition, increased expression of *IL15*, and *HMOX1*, produced by phagocytic cells, suggests enhanced T cell proliferation and activation, correlating with the higher *CD69* levels. These immune responses could prepare the chicks for specific immunity or deal with bacterial challenges.^30,31^ Indeed, IL15, a key cytokine for activation and proliferation of T and NK cells, induces the expression of *CD69* to fight mucosal pathogens. However, evidence suggests that *CD69* exerts a complex immune-regulatory role and could be involved with *HMOX1* and SOCS1 in sustaining an anti-inflammatory response.^32,33^ In conclusion, in these chicks we have both stimulation of the immune response and regulation of this activation. In contrast, in ‘Ctrl’ chicks that did not receive Mix4, we observed higher expression of genes known to drive a pro-inflammatory response including tumor necrosis factor (*TNF*) response (*TNFRSF1A*, *CSF2RA*), phagocyte recruitment and activation (*CSF2RA*, *CEBPB, CCR2*) and T cell differentiation (*CEBPB*, *CD28*, *STAT6*).^34,35^ In addition, Mix4 inoculation exhibited long-term effects, with six genes remaining differentially expressed at 20 days of age. The most important long-term effect of Mix4 appears to be the stimulation of Gallinacin-6 (*GAL6*), also known as AvBD9, a beta-defensin with potent antimicrobial activity against food-borne pathogens.^36,37^ Conversely, lower expression levels of *CD180*, a toll-like receptor homolog and *CD86*, an activation marker of immune cells, suggest a reduced capacity of the control group to effectively respond to pathogens and maintain immune homeostasis compared to the Mix4 treated group.^38^

### Salmonella *Enteritidis infection differentially modulates the systemic immune response dependent on Mix4 inoculation*

Blood analyses revealed that only a small number of genes (8 out of 74, or 11%) showed a modulation of log 2 fold change greater than 2 in infected chicks compared to uninfected chicks (Table 6). Surprisingly, *S*. Enteritidis infection had a less pronounced effect on gene expression than Mix4 inoculation (Table 5), both in terms of the number and magnitude of changes. Furthermore, the same genes (*MX1* and *IL15*) were upregulated in chicks infected by *S*. Enteritidis (at 11 DoA) or inoculated with Mix4 (at 4 DoA) compared to control chicks. The most significant modulation occurred at 7 days post infection (Dpi) or 14 days of age (DoA), with upregulation of 6 genes in infected chicks, suggesting a peak in the type I interferon response (*MX1*), accompanied by a pro-inflammatory response and T cell differentiation (*IL18*, *CEBPB*, *CD28*, *IL15*). Regulatory immune responses were observed at 20 DoA, characterized by increased *SOC3* and decreased *CASP1* transcriptional levels in infected chicks. Notably, systemic immune responses showed minimal changes at 20 and 27 DoA (2 and 3 weeks post-infection), despite very high levels of *Salmonella*.

**Table 6.** Relative gene expression in blood of chicks infected or not with *Salmonella* Enteritidis.

Gene	FC at 11 DoA	FC at 14 DoA	FC at 20 DoA	FC at 27 DoA
*Mx1*	−4.8	−10.5	−2.9	
*IL15*	−2.3	−2.1		
*SOCS3*		−2.0	−2.8	
*IL18*		−3.0		
*CEBPB*		−2.3		
*CD28*		−2.0		
*CASP1*			3.5	
*CTLA4*				−2.4

FC represents the ratio between ‘Ctrl’ and ‘+SE’ groups. Negative FC means that the gene is over-expressed in infected chicks. Ratio > 2.0, with a *p* value < 0.05 were considered.

When chicks were inoculated with Mix4 prior to infection, *Salmonella* infection did not significantly stimulate the systemic immune response. However, more genes were modified by *Salmonella* infection when chicks had received Mix4 compared to infection in chicks that did not receive Mix4 (15 DE genes vs 8 (Tables 6 and 7)). The gene expression changes in the Mix4-treated chicks indicated a dual response: an initial pro-inflammatory response characterized by upregulation of *IL18*, *LGALS3*, *TNFRSF1A*, and *LITAF*, together with Major Histocompatibility Complex class I and II genes (*BF2* and *BLB21)* which help the chicken’s immune system to recognize and respond to pathogens, ensuring both adaptive and innate immune responses. This pro-inflammatory response was accompanied by a regulatory response involving genes such as *IL10*, S*OCS3* and *CISH*, detectable as early as 4 Dpi (Table 7). Interestingly, the same genes (*SOCS3*, *IL18*, *CEBP*), which were upregulated at 7 Dpi in chicks infected without Mix4, were upregulated at 4 Dpi in chicks infected in the presence of Mix4. Despite this, the “Mix4+SE” group did not show higher stimulation of immune genes than the “Mix4” group. Only *IL12B*, encoding a key regulator of Th1 polarization, and the antimicrobial peptide genes *AVBD2* and *AVBD6* were modulated 20 days after *Salmonella* infection. In addition, the expression of *CD180* and *CD86* increased in the “Mix4+SE” group showing that the downregulation of these genes by Mix4 was counterbalanced by *S*. Enteritidis infection, enhancing the ability of the “Mix4+SE” group to respond effectively to pathogens compared to the “Mix4” group.

**Table 7.** Relative gene expression in blood of chicks treated with the Mix4 and infected or not with *Salmonella* Enteritidis.

Gene	FC at 11 DoA	FC at 14 DoA	FC at 20 DoA	FC at 27 DoA
*TNFRSF1A*	−2.4	−2.2		
*IL10*	−3.8			
*SOCS3*	−3.1			
*CISH*	−2.7			
*CEBPB*	−2.1			
*BF2*		−2.9		
*IL18*		−2.8		
*CD180*		−2.5		−2.2
*BLB21*		−2.2		
*LGALS3*		−2.2		
*LITAF*		−2.1		
*CD86*				−2.4
*AVBD2*				2.5
*AVBD6*				2.7
*IL12B*				2.6

FC represents the ratio between ‘+Mix4’ and ‘+Mix4+SE’ groups. Negative FC means that the gene is over-expressed in infected chicks. Ratio > 2.0, with a *p* value < 0.05 were considered.

Overall, Mix4 inoculation before infection appears to modulate the timing and magnitude of the immune response, leading to earlier and more regulated systemic immune activation after infection. These results suggest that Mix4 affects the immune response in the long term, with even more significant changes observed at 6 days of age, just prior to *S*. Enteritidis infection. However, the effect of Mix4 on immune stimulation is partly similar and therefore masked by infection, as only five genes were detected as differentially modulated between chicks treated or not with Mix4 and then infected with *S*. Enteritidis (Supplementary Table SIII). Notably, in the absence of Mix4, we observed a greater inflammatory response as evidenced by higher *CASP1* expression from 4 DoA and a reduced response to antimicrobial peptides (*GAL2* and *GAL6*). In summary, these analyses suggest that differences in infection outcomes within and between groups may be influenced by the immune status of the chicks prior to infection.

### *Difference in the level of* Salmonella *excretion can be related to differences in immune response before and after infection*

As variations in infection outcomes could be influenced by the immune status of chicks prior to infection, we further investigated the immune response in relation to the fate of *Salmonella* colonization using our groups of chicks categorized as short- and long-term super-shedders (SLT-SS), short-term super-shedders (ST-SS), and short- and long-term low shedders (SLT-LS). Prior to infection, chicks that would become SLT-LS exhibited a more robust immune response compared to those that would become SLT-SS (Table 8). This higher activation of immune cells is characterized by increased expression of chemokine receptors such as *CCR2*, which is crucial for activation and recruitment of monocytes/macrophages and *CCR8*, which is important for T lymphocyte recruitment^39^. Additionally, SLT-LS chicks showed higher Galectin 3 expression (*LGALS3*), which activates the NLRP3 inflammasome and trigger the release of the pro-inflammatory cytokine IL18 whose gene expression is also induced. These immune differences were observed as early as 4 DoA, i.e. after Mix4 inoculation, although no significant log 2 fold change greater than 2 was detected at 6 DoA (Table 8). The fact that no gene was differentially expressed between the SLT-SS and SLT-LS groups at 6 DoA, although numerous differences were observed at this time between chicks from isolators that had or had not received Mix4 (Table 5), suggests that Mix4 induces a profound change in the immune response early after its inoculation. However, this effect was not correlated with the level of colonization measured by comparing at 6 DoA the SLT-SS and SLT-LS statuses. In fact, the SLT-SS group included animals that had or had not received Mix4, while all chicks of the SLT-LS group had received the Mix4. This result confirms that the immune response induced by Mix4 inoculation is involved in the low-shedder phenotype.

**Table 8.** Relative gene expression in blood of chicks classified according to their *Salmonella* excretion level.

Day of age	4		6		11		14		20		27	
	Immune gene	Fold change*	Immune gene	Fold change	Immune gene	Fold change	Immune gene	Fold change	Immune gene	Fold change	Immune gene	Fold change
SLT-SS/ST-SS	*IL18*	2.0			*GAL6*	2.6						
					*IL10*	2.9						
					*ARG2*	3.4						
SLT-SS/SLT-LS	*CCR2*	2.4			*TNFRSF1A*	2.1	BLB21	−2.0	SERPINB1	2.6	CD86	−2.5
	*IL18*	2.4			*CCR2*	2.0			TLR5	2.9		
	*LGALS3*	2.4										
	*CCR8*	3.9										
ST-SS/SLT-LS					*ARG2*	−4.3						
					*CD14*	−2.0						

**SLT-SS**= short- and long-term super shedders; **ST-SS**= short-term super shedders; **SLT-LS**= short- and long-term low shedder.

*Positive fold change means that gene is more expressed in the denominator of the ratio. Ratio > 2.0, with a *p* value < 0.05 were considered.

Four days after infection, the genes of chemokine receptors *CCR2* and of TNF receptor 1 (*TNFRSF1A*) both involved in cell recruitment and orchestration of pro-inflammatory immune responses, were more highly expressed in the SLT-LS group compared to SLT-SS. This suggests that the low level of colonization is linked to a more rapid immune response in animals already primed with Mix4. Subsequently, we observed a greater cell mediated immune response in the SLT-SS group characterized by higher *BLB-21* gene expression at 14 DoA and *CD86* at 27 DoA. In contrast, the SLT-LS chickens concomitantly showed a higher anti- and pro-inflammatory response characterized by *SERPINB1* and *TLR5*, respectively. When we analyzed how certain chicks can hinder *Salmonella* multiplication in the long term (comparison between short- and long-term super-shedders (SLT-SS) with short-term super-shedders (ST-SS)) we observed that after *Salmonella* infection, chickens in the ST-SS group exhibited a higher expression of antimicrobial peptide (*GAL6*) and develop an anti-inflammatory micro-environment marked by a higher gene expression of *IL10* and *ARG2*. Indeed, *IL-10* is a crucial cytokine in the immune system, known for its anti-inflammatory properties, whereas *ARG2*, whose expression is regulated by IL-10, downregulate inflammatory mediators.^40,41^

In conclusion, our results suggest that animals that control *Salmonella* infection from the outset exhibited early immune activation, before infection, likely induced by Mix4. This activation is maintained shortly after *Salmonella* infection. Conversely, animals that control *Salmonella* infection at a later stage are able to mount an anti-inflammatory response post infection. However, the difference in *Salmonella* colonization may also be related to GM composition and at least to a close interaction between these two components of the barrier effect.

### The immune gene expression and gut microbial composition are partly correlated

In samples collected in ‘SE’ and ‘+Mix4+SE’ isolators, we tested correlations between immune gene expression levels (dCt) and taxon abundances to determine whether we could identify the bacteria that might be responsible for the type of immune response detected, bearing in mind that correlation is not equivalent to demonstration. For this, we kept the 132 individuals across all timepoints fully characterized for their gut microbial composition and 66 immune genes expression patterns. This was done within the SLT-LS (*n* = 35), SLT-SS (*n* = 61) and ST-SS (*n* = 36) categories at each time point. All these correlations can be downloaded from a database available at https://doi.org/10.57745/YD3IEX.

We identified numerous correlations at 4 DoA in the three categories (Supplementary Table SIV). For example, in the SLT-SS category, *CD25* expression, which is highly expressed in regulatory T cells (which suppress immune responses)^42^, is correlated with the presence of *Ruminococcus* torques group. Furthermore, in this category and at this age *IL10* expression, which is a crucial anti-inflammatory cytokine, is correlated to 26 OTU. These correlations suggested that some commensal bacteria can be correlated to the establishment of immune tolerance or a strong immune cell recruitment. In contrast, different types of correlations were observed in the SLT-LS category at 4 DoA. We especially detected a correlation of *CSF2RA* with *Escherichia*, TNFRSF1A and TLR1 with Clostridiaceae *1* and of GATA3 with Peptostreptococcaceae. Interestingly, these genes contribute to balance immune responses between inflammation and prevention of excessive inflammation, suggesting a stronger stimulation of the immune response compared with the SLT-SS category.

In addition to these 81 correlations detected at 4 DoA within the three categories, it is interesting to note that only three correlations for these three categories were detected at 6 days. Furthermore, after infection, it is important to note that 109 correlations were detected in the SLT-LS category and none in the SLT-SS category. For example, at 11 DoA (4 days post infection), *TLR4* and *CSF2RA* expressions were correlated to Enterococcus and *IL18* (a pro-inflammatory cytokine known for its ability to enhance the production of interferon-gamma) to *Salmonella*.

These correlations therefore confirm the interrelationship between the immune response and the composition of the microbiota. They also highlight the fact that these relationships can have a strong influence on *Salmonella* colonization. This is supported by the correlations observed, before infection, in the SLT-LS category, where all the chicks received Mix4 and where a correlation was found with the bacterial taxa present in the Mix4, i.e. *Escherichia* and Clostridiaceae.

## Discussion

Although most studies of the virulence mechanisms of intestinal pathogens have focused on pathogen–host interactions, it is becoming increasingly clear that the gut microbiota is an essential partner to take into account^43^. Similarly, it appears that there is heterogeneity in infection, the origins of which are poorly understood^5^. Genetic variability of the host was the first variability factor described. However, several studies have pointed out the heterogeneity of *Salmonella* infection and shedding patterns, even when analyzed in inbred hosts.^14,16^ Conversely, numerous articles have shown that differences in bacterial gene content or expression could be attributed to the different levels of infection observed with different *Salmonella* strains.^5,16^ However, our study shows that the super- and low-shedding phenotypes can also be observed using the same *Salmonella* strain and that this cannot be explained by a loss of virulence of the strain during animal infection. We demonstrated, indeed, that the adhesion, invasion, and intracellular multiplication capabilities of *Salmonella* strains recovered from low and super-shedder chickens 3 weeks after infection remained unchanged when compared to the original inoculated strain. These abilities were consistent in both phagocytic and non-phagocytic cells. Therefore, we focused our analysis on the role of other contributing factors.

Several studies on the early stages of *Salmonella* infection show that colonization of the gastrointestinal tract always occurs in a broad context, involving the immune response that is shaped by the host’s specific intestinal microbiota. Moreover, recent researches underscore the gut microbiota (GM) as critical factors in resistance to *Salmonella* colonization,^44^ with well-documented roles in the appearance of the super- and low-shedder phenotypes in mice^45^. In particular, we have previously demonstrated that several pre-infection microbial features within the GM, including the presence of *Enterococcus faecium*, can determine the occurrence of these phenotypes. Consistent with this idea, early inoculation of chicks with a cocktail of four commensal bacteria, which includes *Enterococcus* strain, significantly reduced *Salmonella* excretion levels^18^. However, the exact mechanism has not been fully elucidated, especially in light of the complex interrelations between the microbiota and the immune response. In addition, enteric pathogens such as *Salmonella* can also alter the gut microbiota composition, which in turn modifies the immune response and microbiota composition.

In this study, we demonstrated that *Salmonella* infection altered microbiota composition, without affecting its richness and equitability, as measured with the Chao1 and the Shannon indices and as previously described.^46^ Interestingly, as previously observed, the abundance of facultative anaerobes belonging to the Enterobacteriaceae increased after *Salmonella* infection.^47^ Similarly, our study shows that inoculation of four commensal bacteria at birth does not alter α-diversity but changes β-diversity not only in the short term (a few days after inoculation) but also in the long term. This effect was observed in chicks whether or not they were infected with *Salmonella*.

The protective role of Mix4 inoculation appears to be multifaceted, with a significant effect on both the immune response and gut microbiota composition as well as the metabolic pathways potentially used by the microbiota. Notably, some of these pathways are known to be involved in *Salmonella* colonization as the production of short-chain fatty acids (SCFA), which contributes to create a hostile environment for *Salmonella*, support beneficial gut bacteria, and strengthen gut immune defenses.^48^ Mix4 inoculation also seems to hinder the establishment of a microbiota in anaerobic respiration, which is essential for *Salmonella* to colonize the gut effectively, supporting its growth, enhancing its virulence, and helping it to overcome the colonization resistance posed by the gut microbiota.^49,50^ This result may explain the difference in susceptibility of the animals to *Salmonella*, but more generally, it suggests that it is possible, by inoculation once at birth, to pilot the intestinal microbiota over the long term to confer the desired properties, bearing in mind the importance of the environmental microbiota in the evolution of gut microbiota. This can easily be explained by the fact that a start-up microbiota creates a given ecological niche which guides the implantation of other environmental bacteria.^51^

Our analysis of the immune response also reveals the long-term impact of Mix4 inoculation. However, this impact is more pronounced at 6 and 11 days of age. Thus, in chicks inoculated with Mix4 we observed, compared to the control group, a stimulation of the type 1 interferon response (MX1 and MDA5) and higher gene expression levels for IL15, CD69, HMOX1 and SOCS1. These interconnected genes play a crucial role in T cell proliferation and activation^30^ but also signal the establishment of a regulation of the immune response. This balanced immune response may be linked to the microbiota present at 6 days, but also to the inoculation of the four bacteria making up Mix4, which have these activating and regulating effects. While *lactobacillus* and *clostridium* have an anti-inflammatory effect via the production of SCFA, inoculation of *Enterococcus* and *E. coli* Nissle can have both a pro- and an anti-inflammatory effect, as has been described.^52,53^ Conversely, in the control group, we noted a pro-inflammatory response with an enhanced TNF-related response and increased expression of genes linked to phagocytic activity and recruitment, as well as T cell differentiation. These differences, observed just prior to infection (6 DoA), could contribute to a higher inflammatory response in the absence of the Mix4, which could be favorable to *Salmonella* colonization, as discussed below.

Surprisingly, the impact of *S*. Enteritidis infection on blood immune parameters seemed less pronounced than that of Mix4 inoculation regarding the level and number of immune genes significantly modified (compare Tables 5 and 6). The key distinction lies in the timing: immune gene expressions related to Mix4 were detected at 4 and 6 DoA, while those related to infection are detected at 11 and 14 DoA. This timing discrepancy arises from the different inoculation days, with Mix4 administered at 1 DoA and *Salmonella* at 7 DoA. Therefore, contrary to what was observed in mice, inoculation of chicks with four commensal bacteria induces an immune response in part similar to infection with *S*. Enteritidis. This finding is further supported by the subtle changes in immune response observed at the peak of *Salmonella* colonization (20 days) or later compared to non-infected chicks. This low immune response can be explained by the observation that activation of immune response to *Salmonella* is detected in blood and not in secondary lymphoid organ.^54^ Another explanation could be that in chick, where the infection is asymptomatic, *Salmonella* does not strongly stimulate the immune response as previously described with this low infectious dose^55^ and in the same way as a commensal bacteria during the maturation of the immune response in chicks. Notably, the immune response differs between infected or uninfected chicks when they have previously received Mix4 or not (comparison of genes in Tables 6 and 7). This can be explained by the fact that the innate immune response differs between primary and secondary bacterial infection.^56^ In our study, inoculation with *Salmonella* or Mix4 seems to elicit a primary innate immune response, whereas *Salmonella* infection following Mix4 inoculation would be considered by the immune response as a secondary infection. A more detailed study of immune response would allow us to consolidate this hypothesis.

The heterogeneity of infection is based on the observation that within a chick population or within an isolator, the gut microbiota and immune response of the animals differ from one animal to another leading to the super- and low-shedder phenotypes. As immune response, GM composition and *Salmonella* excretion depend on animal age and the days post infection, we have performed a Ward classification taking into account the level of *Salmonella* in the short and long term. With this classification, we observed, that the short- and long-term low shedders (SLT-LS) only included chicks that received the Mix4, whereas short- and long-term super shedders (SLT-SS), included a large majority of chicks that did not receive the Mix4 (24 out of 32). This analysis clearly shows the protective effect, in certain animals, of the Mix4 against *Salmonella* implantation. However, some chicks that received the Mix4 became SLT-SS, strengthening the heterogeneity of infection. We therefore analyzed the data according to levels of *Salmonella* rather than treatment.

When we analyzed the GM composition between SLT-LS and SLT-SS, there were no differences at 4 DoA but many significant differences at 6 DOA (Table 4). The plausible hypothesis to explain this result is that the differences, obtained when we compared the GM compositions in Table 3, are linked both to the isolator effect (two different isolators) and to differences related to Mix4 inoculation, independently of its action on *Salmonella* colonization. When we merged the data from the two isolators to define the SLT-LS and SLT-SS phenotypes, we discarded the isolator effect and retained only those differences that influenced *Salmonella* colonization, taking into consideration the protective action induced (or not) by Mix4. Consequently, only differences in GM at 6 DOA are likely to be relevant for *Salmonella* colonization.

When we analyzed the immune response between SLT-LS and SLT-SS, differences were observed at 4 DoA but not at 6 DoA (Table 8). By applying the same rationale as with the microbiota, where the merging of the two isolators allows us to take into account only the immune differences influencing *Salmonella* colonization, we can reasonably conclude that the differences in immune response at 4 DoA are essential for *Salmonella* colonization.

Taken together, these results suggest that Mix4 inoculation at 1 DoA modifies, in certain chicks, the immune response at 4 DoA, which subsequently modifies the GM composition at 6 DoA, and may decrease *Salmonella* colonization 4 days post infection (11 DoA). This hypothesis is strongly supported by the numerous correlations we measured at 4 days after Mix4 inoculation (and not at 6 days) between immune gene expression levels and taxon abundances. Thus, it seems that at 4 DoA, inoculation of Mix4 into animals that block *Salmonella* colonization induces a more stimulated immune response, unlike animals that promote colonization and have a more tolerogenic immune response for not responding to the microbiota. This activity could be direct since we detected correlations between *CSF2RA*, *TNFRSF1A* and *TLR1* gene expression and abundance of *Escherichia* and *Clostridium* sensu stricto 1, two genera present in the Mix4 (*Clostridium butyricum* belonging to the *Clostridium* sensu stricto 1 genus and Clostridiaceae *1* family).

Finally, our results suggested how modification of GM composition may inhibit or increase *Salmonella* colonization. The GM metabolic functions identified before infection indicate that the microbiota of SLT-SS display metabolic pathways related to anaerobic respiration and especially those involved in the menaquinol and ubiquinol biosynthesis pathways. This means that the GM of chicks becoming SLT-SS use electron acceptors known to be liberated during an inflammatory response, whereas the GM of chicks becoming SLT-LS are mainly in fermentation by using the degradation of aromatic compounds pathway. This hypothesis is strongly supported by the articles describing, in mice, how *Salmonella* can overcome colonization resistance by using its virulence factors able to trigger intestinal inflammation, which in turn, increases availability of host-derived resources, such as oxygen and nitrate radicals, tetrathionate, and lactate, all together enabling the pathogen to overcome growth inhibition by SCFA.^49,50,57–59^ This hypothesis is also strengthened in our model by the metabolic pathways related to anaerobic respiration, which were enriched (until 20 DoA) in SLT-SS compared to SLT-LS chickens. This hypothesis may explain the higher abundance of Enterobacteriaceae in the infected group compared to non-infected chickens (Table 2). Furthermore, we have shown that animals that received Mix4 but became SLT-SS have a microbiota (before or just after infection) that uses anaerobic respiration like the microbiota of SLT-SS that did not receive Mix4 and unlike the microbiota of SLT-LS that received Mix4. This result confirms the role of the microbiota in heterogeneity of infection, even if we cannot exclude a role for host genetics as we used in this study an immediate inbred chicken line, not a highly inbred line.

From a synthetic point of view, we can conclude that Mix4 inoculation promotes the activation and maturation of the immune response, in certain animals from 4 days, which modifies the evolution of the intestinal microbiota and leads at 6 days to a predominantly fermentative microbiota which has a significant barrier effect, limiting the implantation of *Salmonella* at 7 days and later. Conversely, in control animals and some animals that received Mix4, the “natural” development of the microbiota leads, at 4 DoA, to TNF-driven inflammatory response and to the implantation at 6 DoA of a microbiota using anaerobic respiration, which facilitates implantation and growth of *Salmonella*. After *S*. Enteritidis infection, our results show that chickens already primed with Mix4 exhibit a greater and more rapid pro-inflammatory response, which may explain the metabolic change in the GM, which is more in anaerobic respiration in SLT-LS than in SLT-SS at 20 DoA.

This view is consistent with that described in mice, where *Salmonella* virulence factors trigger an inflammatory response that allows *Salmonella* to use anaerobic respiration and mixed acid fermentation to outcompete the gut microbiota.^58,60^ Thus, many articles describe how *Salmonella* can break the colonization resistance developed by the holobiont. Our work goes a step further by showing that although *Salmonella* is capable of overcoming the barrier effect, even in livestock, this is only true in certain animals in this population. We have shown indeed that there is inter-individual variation in the ability to develop resistance to effective colonization, even before the arrival of *Salmonella*. *Salmonella’s* virulence could therefore only be expressed in certain hosts, and it is possible to make the host resistant to the mechanisms usually put in place by *Salmonella* to overcome the barrier effect. Moreover, our results show that it is the metabolic functions carried out by the microbiota and the immune status of the host that determine the barrier effect. This new dogma refers to the concept of microbiota-nourishing immunity, a host-microbe chimera composed of the microbiota and host factors that confers colonization resistance against pathogens.^61^ By implanting several commensal bacteria at birth, we can trigger a long-term effect on the immune response and the composition of the intestinal microbiota. If the right bacteria are selected, this strategy opens up numerous avenues for controlling not only the barrier effect but also animal performance and behavior, which are driven by these factors.

## Materials and methods

### Ethics approval

The *in vivo* experiment was carried out in compliance with French legislation for the care and use of laboratory animals, after authorization by the French Ministry of Higher Education and Research (permit number APAFIS#5833‐20l60624l6362298 v3).

### Housing conditions

The chicks used in the present study were reared in isolators. An isolator is an experimental breeding system allowing reduced cross contaminations among animals through a constant filtration of air and sterilization of feces (see^15^ for more details), and a control of diet and environmental contamination. A total of 140 white leghorn chicks (PA12 lineage) were raised in this study. PA12 chickens correspond to an immediate inbred chicken line^15^. Moreover, a genotyping performed in 2012 as described by chazara 2013^62^ reveals that the PA12 line, although not considered as highly inbred, is mainly of the B21 MHC haplotype with a small proportion of chickens with B19 MHC haplotype (97% and 3%, respectively). They originated from the specific-pathogen-free (SPF) flock of the PFIE (INRAE Val de Loire, France) which is a core facility specialized in experimental animal infections. The *Salmonella*-free status of chicks before the experiment was confirmed by analyzing blood and fecal samples. Chicks were fed ad libitum, had free access to drinking water, and a 12:12 L:D lighting scheme was applied. The two isolators where the birds had been infected were in one room, while the uninfected birds were in another.

### Bacterial strains and culture conditions

A streptomycin and nalidixic acid-resistant *Salmonella* Enteritidis phage type 4 strain LA5 was cultured aerobically in trypticase soya broth (TSB; BioMérieux) supplemented with 500 μg/ml of streptomycin (Sigma‐Aldrich) for 24 h at 37°C with shaking. The culture was harvested by centrifugation at 4500 × g for 20 min at room temperature and suspended in phosphate buffered saline (PBS) containing 50% glycerol. The bacterial suspension was aliquoted and stored at −80°C. On the inoculation day, the challenge inoculum was prepared by diluting a *S*. Enteriditis freezed aliquot in PBS to achieve a final viable cell concentration of 2.5 × 10^5^ CFU/ml.

A mix (namely “Mix4”) of four commensal bacteria was developed based on the literature and our previous results and has been already described^18^. *Escherichia coli* Nissle 1917,^63^ *Lactobacillus rhamnosus* strain DSM 7133, *Clostridium butyricum* strain DSM 10,702 and *Enterococcus faecium* DSM 7134 strains were cultured independently and mixed just before oral inoculation. The inoculum was prepared by mixing (1) 2.5 mL of an overnight culture of *E. coli* Nissle 1917 strain grown in 10 ml BHI medium (Difco) at 37°C without agitation (2) 2.5 mL of an overnight culture of *E. faecium* strain grown in 10 ml BHI medium at 37°C without agitation (3) 2.5 mL of an overnight culture of *L. rhamnosus* strain grown in 10 ml BHI medium at 37°C into an anaerobic jar with gas pack CO2 gas generator (BD BBL) (4) 2.5 mL of a one-day culture of *C. butyricum* strain grown in Wilkins Chalgren medium at 37°C. The bacterial numbers of *E. coli*, *L. rhamnosus* and *E. faecium* in the inoculum were determined by plating serial dilution counting on (1) Tryptic Soy Agar (TSA; Bio-Rad, Marnes-la-Coquette, France) (2) *m-Enterococcus* (Difco) (3) DeMan-Rogosa-Sharpe agar (MRS; Bio-Rad, Marnes-la-Coquette, France), respectively. The number of *C. butyricum* was determined by counting colonies in bacterial cultures made on a TSA medium containing, respectively, ammonium citrate (0.5 g/L) and sodium metabisulphite (1 g/L) at 37°C for 48 h MRS plates were incubated anaerobically into a jar with gas pack CO2 gas generator.

### Eukaryotic cells and culture conditions

Cell lines from different species were tested: chicken hepatocellular carcinoma LMH (ATCC CRL-2117), new-born piglet intestinal IPEC-1^64^ for epithelial cells and macrophage-like chicken cell-line HD11^65^ and parental porcine monomyeloid cell line, 3D4/2^66^ for macrophage cells. Cells were routinely grown in 75 cm^2^ plastic tissue culture flasks at 37°C under 5% CO2 in the different recommended cell culture media without antimicrobial compounds.

## Methods details

### Adhesion and invasion assays

Cells were cultured for 5 days in 24-well tissue culture plates (Falcon) to obtain subconfluent monolayers. Gentamicin protection assays were performed as described previously.^67^ Each experiment used three plates for adhesion, entry and intracellular multiplication steps. In all conditions, cells were infected with 10^7^ CFU of the different *Salmonella* strains diluted in 300 μL of DMEM without serum at a MOI = 10 (multiplicity of infection). For adhesion assays, after 60 min of bacteria-cell contact at 37°C, cells were washed at least four times with PBS (phosphate buffer saline, Sigma) and then lysed at 4°C with cold distilled water. Viable bacteria (extra- and intra-cellular) were counted after plating serial dilutions on TSA (Tryptic Soy Agar). The number of internalized bacteria was determined using a gentamicin protection assay to kill extracellular bacteria, as previously described. After 60 min of bacteria-cell contact and 90 min treatment with gentamicin at 100 μg/ml (Gibco) to kill extracellular bacteria, cells were washed and lysed in cold distilled water. The number of internalized bacteria was enumerated as before. To measure the intracellular multiplication, after 90 min treatment with gentamicin at 100 μg/ml (Gibco), cells were washed and incubated for 18 h with 10 μg/ml gentamicin at 37°C. Cells were then washed one time and lysed at 4°C with cold distilled water. Intracellular viable bacteria were counted after plating serial dilutions on TSA (Tryptic Soy Agar). Results were expressed as the mean ± SEM of the number of adhered or invaded or multiplied bacteria relative to 10^7^ inoculated CFU. Experiments were performed in duplicate and repeated at least three times for each strain by two different people.

### In vivo experiment

For the *in vivo* experiment, the isolators were cleaned but not sterilized and were left open to receive the environmental microbiota. Four treatment groups of 35 chicks were randomly constituted and housed in 4 different isolators (designated by isolator ‘+Mix4’, ‘Ctrl’, ‘+Mix4+SE’ and ‘+SE’). On the day of hatching the ‘+Mix4’ and ‘+Mix4+SE’ groups were orally inoculated with 200 μl of the commensal bacteria containing 7.7 × 10^6^ CFU of *E. coli* Nissle 1917, 1.1 × 10^7^ CFU of *E. faecium*, 1.3 × 10^3^ CFU of *C. butyricum*, and 2.5 × 10^6^ CFU of *L. rhamnosus*. The isolators were then closed. At 7 days of age, the ‘+Mix4+SE’ and ‘+SE’ groups were orally challenged with 5 × 10^4^ CFU of *S*. Enteritidis LA5 (i.e. 0.2 mL of a solution containing 2.5 × 10^5^ CFU/mL); animals of the control groups received the same volume of a sterile saline solution.

Seven chicks for the ‘Ctrl’ and ‘+Mix4’ groups, and 30 chicks for the ‘+Mix4+SE’ and ‘+SE’ groups were randomly selected for blood sampling and feces collection at 4 and 6 days of age (DoA) and at 4, 7, 13 and 20 days post infection (dpi). Fecal samples were collected by gently pressing the chick’s abdomen and were rapidly frozen in a dry ice/alcohol bath for microbiota analysis, or in ice for bacterial numeration.^22^ At 21 dpi, animals were euthanized by carbon dioxide inhalation. Venous occipital sinus blood samples (100 μl) were collected to perform real-time PCR. Blood were then mixed with 100 μl PBS and 1.2 ml Trizol to be directly frozen at −80°C until RNA extraction.

### Bacteriology

To determine the bacterial load, the fecal samples were weighted and homogenized in TSB medium (ThermoFisher Scientific, Illkirch-Graffenstaden, France). CFU/g feces were determined by plating serial 10-fold dilutions on *Salmonella*–*Shigella* agar plates containing 500 μg/mL streptomycin. When necessary, the sample contents were enriched in 30 ml TSB to reveal contamination below the detection threshold. After 24 h at 37°C, these cultures were plated on *Salmonella*–*Shigella* medium containing streptomycin and incubated for 24 h. After enrichment, the detection threshold was one bacterium per organ.

### Immune gene expression

Total RNA were extracted from blood samples using the Nucleospin 8 RNA Kit (Macherey Nagel, Düren, Germany) as recommended by the manufacturer’s protocol and stored at −80°C. RNA purity and concentration were measured with a Nanodrop (Thermoscientific Nanodrop, Illkirch-Graffenstaden, France) and their integrity was assessed by 1% agarose gel electrophoresis. 200 ng of RNA from each sample were reverse-transcribed using iScriptTM cDNA Synthesis kit (Promega, Charbonnières-les-Bains, France) as described by the manufacturer and the cDNAs obtained were stored at −20°C and diluted just before use.

The primer sequences used in this study have been partly obtained by Fluidigm D3TM (Fluidigm France, Les Ulis, France) assay design service (Supplementary Table SIII). The specificity of the primers was checked by melting curve analysis and gel electrophoresis of the amplified product (data not shown). PCR efficiencies of the assays were determined via calibration curves with a 5-point dilution series of pooled blood samples from the experiment in duplicates.

High-throughput qPCR was carried out using the BioMark-HD 96.96 dynamic array IFC chip (Fluidigm Corporation, CA, USA). Briefly, after a pre-amplification step ensuring adequate amounts of templates of the target genes for the qPCR, the samples were treated with Exonuclease I (E. coli) (New England Biolabs Evry, France) to degrade unincorporated primers. Exonuclease I-treated samples were diluted 1:5 with TE buffer and 1.25 μl of each of them used to prepare a Sample Assay as recommended by the manufacturer’s instructions. Likewise, a Primer Assay was prepared. For this, IFC controller (Fluidigm) was used to prime 96.96 dynamic arrays IFC Chip (Fluidigm) with control line fluid. After loading samples and assay mixes in the appropriate inlets, the chip was placed in the BioMark Instrument for PCR at 95°C for 10 min, followed by 40 cycles at 95°C for 15 sec and 60°C for 1 min. After completion of the run, a melting curve of the amplified products was determined to confirm the specificity of the reactions. Data were analyzed with the Fluidigm real-time PCR Analysis Software available in the BioMark instrument (Fluidigm). For normalization, three potential reference genes were selected (GUSB, LDHA, and SDHA). Primers have been designed between two exons of the eukaryotic gene.

### 16S metabarcoding

Microbial DNA was extracted using the QIAamp DNA Stool mini-kit following the manufacturer’s instructions (Qiagen, Hilden, Germany). Briefly, 25 mg of fecal or cecal contents were transferred to a tube containing lysis buffer and sterile zirconium beads. Samples were homogenized at maximum speed (FastPrep FP120, MP Biomedicals) for four cycles of 45 s each, with cooling between the second and the third cycle, and then heated at 70°C for 15 min. Following centrifugation (5 min at 16 000 g, 4°C), a second extraction step was performed. The two supernatants were pooled for the DNA purification step. Proteinase K was added, and the sample was heated at 70°C for 10 min to degrade proteins. Ethanol was then added, and DNA was purified using QIAamp columns as per the manufacturer’s protocol. The sample was eluted in 200 µL of Tris-EDTA buffer AE (Qiagen). DNA quantity and quality were measured with a Nanodrop spectrophotometer and then diluted to a concentration of 5 ng/mL. PCR amplification was performed using the forward primer 5´-*TCGTCGGCAGCGTCAGATGTGTATAAGAGACAG –* MID – GT – CCTACGGGNGGCWGCAG-3´ and reverse primer 5´ - *GTCTCGTGGGCTCGGAGATGTGTATAAGAGACAG –* MID – GT – GACTACHVGGGTATCTAATCC-3´. The sequences in italics served as the index and adapter ligation, while the underlined sequences enabled amplification over the V3/V4 region of 16S rRNA genes. MIDs (Multiplex Identifier) represent different sequences of 5, 6, 9, or 12 base pairs in length, used to differentiate samples within the sequencing pools. GTs correspond to a sequence which separates the barcode from the following sequence which will allow amplification of the V3/V4 region with degenerate bases: *N* = the 4 bases at random, W = A or T. PCR amplification was performed using a HotStarTaq Plus MasterMix kit (Qiagen). The resulting PCR products were purified using AMPure beads (Beckman Coulter). Next, the concentration of PCR products was determined using spectrophotometry, and the DNA was diluted to 100 ng/µl. Groups of 14 PCR products with different MID sequences were indexed with the same indices using Nextera XT Index Kit, following the manufacturer’s instructions (Illumina, San Diego, CA, USA). Prior to sequencing, the concentration of differently indexed samples was determined using a KAPA Library Quantification Complete kit (Kapa Biosystems, Boston, MA, USA). All indexed samples were diluted to 4 ng/µl, and 20 pM phiX DNA was added to a final concentration of 5% (v/v). Sequencing was performed using MiSeq Reagent Kit v3 and MiSeq apparatus according to the manufacturer’s instructions (Illumina). The16S rRNA gene sequencing included 300 samples collected from 4 groups of chicks bred in separate isolators (see above). The read assembly yielded 13,963,327 16S rRNA gene sequences.

An initial quality-trimming step of the raw reads was performed using TrimmomaticPE 0.30.^68^ The data were then uploaded to the FROGS analysis pipeline for further microbiota characterization.^69^ First, paired-end reads from each sample were clustered with no mismatch in MID sequences. They were then selected based on an expected read size of 292 bp, and a total amplicon size ranging from 350 bp to 550 bp with a mean of 460 bp. The resulting sequences were clustered using Swarm,^70^ with aggregation distance parameters set to 1 and 3 for the denoising and final clustering steps, respectively. OTUs containing chimeric sequences were removed using VSearch.^49^ Additional quality control steps included removal of very rare OTUs (relative abundance < 0.00005% of the total read numbers) and those containing sequences matching phiX sequences recorded in a specific databank.^69^ Finally, the resulting 287 OTUs, totaling 3,578,036 sequences, were classified using an NCBI Blast+ search within the Silva SSU 123 database.^71,72^

### Bioinformatic and statistical analyses

Differences in means between two groups were assessed using Student t-tests. To compare more than two groups, depending on the sample sizes, kind of variable and levels of comparison, we either used one-way ANOVAs, Kruskal Wallis multiple comparison tests, PERMANOVA (9999 permutations). Student t-tests, one-way ANOVA, Kruskall-Wallis tests were performed using the dedicated native R tools (R Development Core Team, 2020); PERMANOVA tests were performed using the adonis() function of the R-package vegan^73^.

Differential abundances were assessed following the hypothesis that abundances in each sample followed negative binomial distributions. Under this scheme, relative abundance may be modeled by fitting a generalized linear model. Significant log 10 fold change ratios were detected using Wald tests and Benjamini–Hochberg adjustment for multiple testing (*p* < 0.01). The computations were performed using the R-package DESeq2 v. 1.24.0^74^.

Multivariate modeling of *Salmonella* relative abundances was performed using linear mixed-effects models, allowing random effects to be taken into account. These computations were performed using R-package nlme^75^.

Diversity assessment was based on the Chao1 and Shannon α-diversity indices and the Bray-Curtis β-diversity index. The computations of diversity indices were performed using R-package phyloseq v. 1.28.00.^76^

Functional gene families and MetaCyc pathways were predicted using the PICRUSt2 package.^26^ MetaCyc pathways were aggregated at the super-pathway level using MetaCyc database^77^. Differential abundances were computed for the pathways included within the Metacyc classes.

Super and low shedders classification was made either directly by hierarchical clustering of the bacterial cell counts at 11, 14, 20, and 27 DoA (Figure 1) or by first averaging the short term (11, 14 DoA) and long term (20, 27 DoA) excretions which were later classified by hierarchical clustering (Figure 6). This second method led to the identification of SLT-SS, SLT-LS, and ST-SS groups.

Correlations between gut microbial species and significantly differentially expressed immune gene were investigated using HAllA^78^. Briefly, HAllA is a tool allowing to search for significant relationships among high-dimensionality, heterogeneous datasets using a hierarchical false discovery correction procedure. A false discovery rate of 0.01 using Benjamini – Hochberg – Yekutieli (BHY) correction was used to screen for candidate correlations.

## Supplementary Material

Supplemental Material

## Data Availability

The accession number for raw 16S rRNA gene sequencing data reported in this paper is deposited in the Sequence Read Archive (SRA) of the European Nucleotide Archive (ENA) (PRJEB39111). All the correlations between immune response and microbiota composition can be downloaded in an Excel file at https://doi.org/10.57745/YD3IEX.

## References

[1] European Food Safety, A. European Centre for Disease, P. Control. The European Union One Health 2022 zoonoses report. Efsa J. 2023;21(12):e8442. doi:10.2903/j.efsa.2023.8442. 38089471 PMC10714251

[2] World Health Organization. WHO bacterial priority pathogens list, 2024: bacterial pathogens of public health importance, to guide research, development, and strategies to prevent and control antimicrobial resistance. Geneva, Switzerland: World Health Organization; 2024.

[3] European Food Safety, A. European Centre for Disease, P. Control. The European Union One Health 2021 zoonoses report. Efsa J. 2022;20(12):e07666. doi:10.2903/j.efsa.2022.7666. 36524203 PMC9745727

[4] Velge P, Cloeckaert A, Barrow P. Emergence of *Salmonella* epidemics: the problems related to *Salmonella enterica* serotype Enteritidis and multiple antibiotic resistance in other major serotypes. Vet Res. 2005;36(3):267–33. doi:10.1051/vetres:2005005. 15845226

[5] Kempf F, La Ragione R, Chirullo B, Schouler C, Velge P. Super shedding in enteric pathogens: a review. Microorganisms. 2022;10(11):2101. doi:10.3390/microorganisms10112101. 36363692 PMC9692634

[6] Woolhouse ME, Dye C, Etard JF, Smith T, Charlwood JD, Garnett GP, Hagan P, Hii JL, Ndhlovu PD, Quinnell RJ, et al. Heterogeneities in the transmission of infectious agents: implications for the design of control programs. Proc Natl Acad Sci USA. 1997;94(1):338–342. doi:10.1073/pnas.94.1.338. 8990210 PMC19338

[7] Kulow MJ, Gonzales TK, Pertzborn KM, Dahm J, Miller BA, Park D, Gautam R, Kaspar CW, Ivanek R, Dopfer D. Differences in colonization and shedding patterns after oral challenge of cattle with three Escherichia coli O157: H7 strains. Appl Environ Microbiol. 2012;78(22):8045–8055. doi:10.1128/AEM.02363-12. 22961893 PMC3485966

[8] Rapp D, Ross CM, Pleydell EJ, Muirhead RW. Differences in the fecal concentrations and genetic diversities of Campylobacter jejuni populations among individual cows in two dairy herds. Appl Environ Microbiol. 2012;78(21):7564–7571. doi:10.1128/AEM.01783-12. 22904055 PMC3485710

[9] Huang TH, Uthe JJ, Bearson SM, Demirkale CY, Nettleton D, Knetter S, Christian C, Ramer-Tait AE, Wannemuehler MJ, Tuggle CK, et al. Distinct peripheral blood RNA responses to *Salmonella* in pigs differing in *Salmonella* shedding levels: intersection of IFNG, TLR and miRNA pathways. PLOS ONE. 2011;6(12):e28768. doi:10.1371/journal.pone.0028768. 22174891 PMC3236216

[10] Kommadath A, Bao H, Arantes AS, Plastow GS, Tuggle CK, Bearson SM, Guan le L, Stothard P. Gene co-expression network analysis identifies porcine genes associated with variation in *Salmonella* shedding. BMC Genomics. 2014;15(1):452. doi:10.1186/1471-2164-15-452. 24912583 PMC4070558

[11] Cazals A, Rau A, Estelle J, Bruneau N, Coville JL, Menanteau P, Rossignol MN, Jardet D, Bevilacqua C, Bed’hom B, et al. Comparative analysis of the caecal tonsil transcriptome in two chicken lines experimentally infected with *Salmonella* Enteritidis. PLOS ONE. 2022;17(8):e0270012. doi:10.1371/journal.pone.0270012. 35976909 PMC9384989

[12] Chaussé AM, Grépinet O, Bottreau E, Le Vern Y, Menanteau P, Trotereau J, Robert V, Wu Z, Kerboeuf D, Beaumont C, Velge P, Fang FC. Expression of Toll-like receptor 4 and downstream effectors in selected cecal cell subpopulations of chicks resistant or susceptible to *Salmonella* carrier state. Infect Immun. 2011;79(8):3445–3454. doi:10.1128/IAI.00025-11. 21628520 PMC3147551

[13] Lawley TD, Bouley DM, Hoy YE, Gerke C, Relman DA, Monack DM. Host transmission of *Salmonella enterica* serovar Typhimurium is controlled by virulence factors and indigenous intestinal microbiota. Infect Immun. 2008;76(1):403–416. doi:10.1128/IAI.01189-07. 17967858 PMC2223630

[14] Cazals A, Estelle J, Bruneau N, Coville JL, Menanteau P, Rossignol MN, Jardet D, Bevilacqua C, Rau A, Bed’hom B, et al. Differences in caecal microbiota composition and *Salmonella* carriage between experimentally infected inbred lines of chickens. Genet Sel Evol. 2022;54(1):7. doi:10.1186/s12711-022-00699-6. 35093028 PMC8801081

[15] Menanteau P, Kempf F, Trotereau J, Virlogeux-Payant I, Gitton E, Dalifard J, Gabriel I, Rychlik I, Velge P. Role of systemic infection, cross contaminations and super-shedders in *Salmonella* carrier state in chicken. Environ Microbiol. 2018;20(9):3246–3260. doi:10.1111/1462-2920.14294. 29921019

[16] Gopinath S, Carden S, Monack D. Shedding light on *Salmonella* carriers. Trends Microbiol. 2012;20(7):320–327. doi:10.1016/j.tim.2012.04.004. 22591832

[17] Kempf F, Cordoni G, Chaussé AM, Drumo R, Brown H, Horton DL, Paboeuf F, Denis M, Velge P, La Ragione R, et al. Inflammatory responses induced by the monophasic variant of *Salmonella* Typhimurium in pigs play a role in the high shedder phenotype and fecal microbiota composition. mSystems. 2023;8(1):e0085222. doi:10.1128/msystems.00852-22. 36629432 PMC9948705

[18] Kempf F, Menanteau P, Rychlik I, Kubasova T, Trotereau J, Virlogeux-Payant I, Schaeffer S, Schouler C, Drumo R, Guitton E, et al. Gut microbiota composition before infection determines the *Salmonella* super- and low-shedder phenotypes in chicken. Microb Biotechnol. 2020;13(5):1611–1630. doi:10.1111/1751-7915.13621. 32639676 PMC7415355

[19] Edelman SM, Kasper DL. Symbiotic commensal bacteria direct maturation of the host immune system. Curr Opin Gastroenterol. 2008;24(6):720–724. doi:10.1097/MOG.0b013e32830c4355. 19122522

[20] Broom LJ, Kogut MH. The role of the gut microbiome in shaping the immune system of chickens. Vet Immunol Immunopathol. 2018;204:44–51. doi:10.1016/j.vetimm.2018.10.002. 30596380

[21] Zenner C, Hitch TCA, Riedel T, Wortmann E, Tiede S, Buhl EM, Abt B, Neuhaus K, Velge P, Overmann J, et al. Early-life immune system maturation in chickens using a synthetic community of cultured gut bacteria. mSystems. 2021 6. 6(3). doi:10.1128/mSystems.01300-20. PMC8269260 34006629

[22] Velge P, Menanteau P, Chaumeil T, Barilleau E, Trotereau J, Virlogeux-Payant I. Two In Vivo Models to Study Salmonella Asymptomatic Carrier State in Chicks. In: Gal-Mor O, editors. Bacterial Virulence: Methods Mol Biol. New York, US: Springer; 2022. Vol. 2427; p. 249–264. 10.1007/978-1-0716-1971-1_20 35619039

[23] Roche SM, Holbert S, Trotereau J, Schaeffer S, Georgeault S, Virlogeux-Payant I, Velge P. *Salmonella* Typhimurium invalidated for the three currently known invasion factors keeps its ability to invade several cell models. Front Cell Infect Microbiol. 2018;8:273. doi:10.3389/fcimb.2018.00273. 30148118 PMC6095967

[24] Crhanova M, Hradecka H, Faldynova M, Matulova M, Havlickova H, Sisak F, Rychlik I. Immune response of chicken gut to natural colonisation by gut microflora and to Salmonella enterica serovar enteritidis infection. Infect. Immun. 2011;79(7):2755–63. doi:10.1128/iai.01375-10. 21555397 PMC3191970

[25] Ballou AL, Ali RA, Mendoza MA, Ellis JC, Hassan HM, Croom WJ, Koci MD. Development of the chick microbiome: how early exposure influences future microbial diversity. Front Vet Sci. 2016;3:2. doi:10.3389/fvets.2016.00002. 26835461 PMC4718982

[26] Douglas GM, Maffei VJ, Zaneveld JR, Yurgel SN, Brown JR, Taylor CM, Huttenhower C, Langille MGI. PICRUSt2 for prediction of metagenome functions. Nat Biotechnol. 2020;38(6):685–688. doi:10.1038/s41587-020-0548-6. 32483366 PMC7365738

[27] Nagao-Kitamoto H, Kitamoto S, Kuffa P, Kamada N. Pathogenic role of the gut microbiota in gastrointestinal diseases. Intest Res. 2016;14(2):127–138. doi:10.5217/ir.2016.14.2.127. 27175113 PMC4863046

[28] Aussel L, Pierrel F, Loiseau L, Lombard M, Fontecave M, Barras F. Biosynthesis and physiology of coenzyme Q in bacteria. Biochim Biophys Acta. 2014;1837(7):1837, 1004–1011. doi:10.1016/j.bbabio.2014.01.015. 24480387

[29] Garrido D, Alber A, Kut E, Chanteloup NK, Lion A, Trotereau A, Dupont J, Tedin K, Kaspers B, Vervelde L, et al. The role of type I interferons (IFNs) in the regulation of chicken macrophage inflammatory response to bacterial challenge. Dev Comp Immunol. 2018;86:156–170. doi:10.1016/j.dci.2018.04.025. 29729283

[30] Jabri B, Abadie V. IL-15 functions as a danger signal to regulate tissue-resident T cells and tissue destruction. Nat Rev Immunol. 2015;15(12):771–783. doi:10.1038/nri3919. 26567920 PMC5079184

[31] Kanegane H, Tosato G. Activation of naive and memory T cells by interleukin-15. Blood. 1996;88(1):230–235. doi:10.1182/blood.V88.1.230.230. 8704178

[32] Gonzalez-Amaro R, Cortes JR, Sanchez-Madrid F, Martin P. Is CD69 an effective brake to control inflammatory diseases? Trends Mol Med. 2013;19(10):625–632. doi:10.1016/j.molmed.2013.07.006. 23954168 PMC4171681

[33] Sebastian VP, Salazar GA, Coronado-Arrazola I, Schultz BM, Vallejos OP, Berkowitz L, Alvarez-Lobos MM, Riedel CA, Kalergis AM, Bueno SM. Heme oxygenase-1 as a modulator of intestinal inflammation development and progression. Front Immunol. 2018;9:1956. doi:10.3389/fimmu.2018.01956. 30258436 PMC6143658

[34] Sanmarco LM, Chao CC, Wang YC, Kenison JE, Li Z, Rone JM, Rejano-Gordillo CM, Polonio CM, Gutierrez-Vazquez C, Piester G, et al. Identification of environmental factors that promote intestinal inflammation. Nature. 2022;611(7937):801–809. doi:10.1038/s41586-022-05308-6. 36266581 PMC9898826

[35] Souza RF, Caetano MAF, Magalhaes HIR, Castelucci P. Study of tumor necrosis factor receptor in the inflammatory bowel disease. World J Gastroenterol. 2023;29(18):2733–2746. doi:10.3748/wjg.v29.i18.2733. 37274062 PMC10237104

[36] Lynn DJ, Higgs R, Lloyd AT, O’Farrelly C, Herve-Grepinet V, Nys Y, Brinkman FS, Yu PL, Soulier A, Kaiser P, et al. Avian beta-defensin nomenclature: a community proposed update. Immunol Lett. 2007;110(1):86–89. doi:10.1016/j.imlet.2007.03.007. 17467809

[37] van Dijk A, Veldhuizen EJ, Kalkhove SI, Tjeerdsma-van Bokhoven JL, Romijn RA, Haagsman HP. The β-Defensin Gallinacin-6 is expressed in the chicken digestive tract and has antimicrobial activity against food-borne pathogens. Antimicrob Agents Chemother. 2007;51(3):912–922. doi:10.1128/AAC.00568-06. 17194828 PMC1803155

[38] Edwards K, Lydyard PM, Kulikova N, Tsertsvadze T, Volpi EV, Chiorazzi N, Porakishvili N. The role of CD180 in hematological malignancies and inflammatory disorders. Mol Med. 2023;29(1):97. doi:10.1186/s10020-023-00682-x. 37460961 PMC10353253

[39] Liu L, Rangan L, Vanalken N, Kong Q, Schlenner S, De Jonghe S, Schols D, Van Loy T. Development of a cellular model to study CCR8 signaling in tumor-infiltrating regulatory T cells. Cancer Immunol Immunother. 2024;73(1):11. doi:10.1007/s00262-023-03607-z. 38231448 PMC10794316

[40] Dowling JK, Afzal R, Gearing LJ, Cervantes-Silva MP, Annett S, Davis GM, De Santi C, Assmann N, Dettmer K, Gough DJ, et al. Mitochondrial arginase-2 is essential for IL-10 metabolic reprogramming of inflammatory macrophages. Nat Commun. 2021;12(1):1460. doi:10.1038/s41467-021-21617-2. 33674584 PMC7936006

[41] Marti ILAA, Reith W. Arginine-dependent immune responses. Cell Mol Life Sci. 2021;78(13):5303–5324. doi:10.1007/s00018-021-03828-4. 34037806 PMC8257534

[42] Ward NC, Yu A, Moro A, Ban Y, Chen X, Hsiung S, Keegan J, Arbanas JM, Loubeau M, Thankappan A, et al. IL-2/CD25: a long-acting fusion protein that promotes immune tolerance by selectively targeting the IL-2 receptor on regulatory T cells. J Immunol. 2018;201(9):2579–2592. doi:10.4049/jimmunol.1800907. 30282751 PMC6200646

[43] Zheng D, Liwinski T, Elinav E. Interaction between microbiota and immunity in health and disease. Cell Res. 2020;30(6):492–506. doi:10.1038/s41422-020-0332-7. 32433595 PMC7264227

[44] Volf J, Faldynova M, Matiasovicova J, Sebkova A, Karasova D, Prikrylova H, Havlickova H, Rychlik I Probiotic mixtures consisting of representatives of bacteroidetes and selenomonadales increase resistance of newly hatched chicks to salmonella enteritidis infection. Microorganisms. 2024;12(11):2145. doi: 10.3390/microorganisms12112145. 39597533 PMC11596081

[45] Ruddle SJ, Massis LM, Cutter AC, Monack DM. *Salmonella*-liberated dietary L-arabinose promotes expansion in superspreaders. Cell Host Microbe. 2023;31(3):405–417 e405. doi:10.1016/j.chom.2023.01.017. 36812913 PMC10016319

[46] Mon KKZ, Zhu Y, Chanthavixay G, Kern C, Zhou H. Integrative analysis of gut microbiome and metabolites revealed novel mechanisms of intestinal *Salmonella* carriage in chicken. Sci Rep. 2020;10(1):4809. doi:10.1038/s41598-020-60892-9. 32179754 PMC7075953

[47] Mon KK, Saelao P, Halstead MM, Chanthavixay G, Chang HC, Garas L, Maga EA, Zhou H. *Salmonella enterica* Serovars Enteritidis Infection Alters the Indigenous Microbiota Diversity in Young Layer Chicks. Front Vet Sci. 2015;2:61. doi:10.3389/fvets.2015.00061. 26664988 PMC4672283

[48] van der Hee B, Wells JM. Microbial regulation of Host physiology by short-chain fatty acids. Trends Microbiol. 2021;29(8):700–712. doi:10.1016/j.tim.2021.02.001. 33674141

[49] Rogers AWL, Tsolis RM, Baumler AJ. *Salmonella* versus the microbiome. Microbiol Mol Biol Rev. 2021;85(1). doi:10.1128/MMBR.00027-19. PMC8549850 33361269

[50] Shelton CD, Yoo W, Shealy NG, Torres TP, Zieba JK, Calcutt MW, Foegeding NJ, Kim D, Kim J, Ryu S, et al. *Salmonella enterica* serovar Typhimurium uses anaerobic respiration to overcome propionate-mediated colonization resistance. Cell Rep. 2022;38(1):110180. doi:10.1016/j.celrep.2021.110180. 34986344 PMC8800556

[51] Baquero F, Coque TM, Galan JC, Martinez JL. The origin of niches and species in the bacterial world. Front Microbiol. 2021;12:657986. doi:10.3389/fmicb.2021.657986. 33815348 PMC8010147

[52] Dai J, Jiang M, Wang X, Lang T, Wan L, Wang J. Human-derived bacterial strains mitigate colitis via modulating gut microbiota and repairing intestinal barrier function in mice. BMC Microbiol. 2024;24(1):96. doi:10.1186/s12866-024-03216-5. 38521930 PMC10960398

[53] Guo S, Chen S, Ma J, Ma Y, Zhu J, Ma Y, Liu Y, Wang P, Pan Y. *Escherichia coli* Nissle 1917 protects intestinal barrier function by inhibiting NF- κ B-Mediated activation of the MLCK-P-MLC signaling pathway. Mediators Inflamm. 2019;2019:1–13. doi:10.1155/2019/5796491. PMC6636522 31354386

[54] McSorley SJ, Asch S, Costalonga M, Reinhardt RL, Jenkins MK. Tracking *Salmonella*-specific CD4 T cells in vivo reveals a local mucosal response to a disseminated infection. Immunity. 2002;16(3):365–377. doi:10.1016/S1074-7613(02)00289-3. 11911822

[55] Sadeyen JR, Trotereau J, Velge P, Marly J, Beaumont C, Barrow PA, Bumstead N, Lalmanach AC. *Salmonella* carrier state in chicken: comparison of expression of immune response genes between susceptible and resistant animals. Microbes Infect. 2004;6(14):1278–1286. doi:10.1016/j.micinf.2004.07.005. 15555534

[56] Kirby AC, Yrlid U, Wick MJ. The innate immune response differs in primary and secondary *Salmonella* infection. J Immunol. 2002;169(8):4450–4459. doi:10.4049/jimmunol.169.8.4450. 12370380

[57] Drumo R, Pesciaroli M, Ruggeri J, Tarantino M, Chirullo B, Pistoia C, Petrucci P, Martinelli N, Moscati L, Manuali E, et al. *Salmonella enterica* serovar Typhimurium exploits inflammation to modify swine intestinal microbiota. Front Cell Infect Microbiol. 2015;5:106. doi:10.3389/fcimb.2015.00106. 26835435 PMC4722131

[58] Winter SE, Thiennimitr P, Winter MG, Butler BP, Huseby DL, Crawford RW, Russell JM, Bevins CL, Adams LG, Tsolis RM, et al. Gut inflammation provides a respiratory electron acceptor for Salmonella. Nature. 2010;467(7314):426–429. doi:10.1038/nature09415. 20864996 PMC2946174

[59] Woelfel S, Silva MS, Stecher B. Intestinal colonization resistance in the context of environmental, host, and microbial determinants. Cell Host Microbe. 2024;32(6):820–836. doi:10.1016/j.chom.2024.05.002. 38870899

[60] Nguyen BD, Sintsova A, Schubert C, Sichert A, Scheidegger C, Naf J, Huttman J, Lentsch V, Keys T, Rutschmann C, et al. *Salmonella* Typhimurium screen identifies shifts in mixed-acid fermentation during gut colonization. Cell Host Microbe. 2024;32(10):1758–1773 e1754. doi:10.1016/j.chom.2024.08.015. 39293436

[61] Litvak Y, Baumler AJ. Microbiota-nourishing immunity: a Guide to understanding our microbial self. Immunity. 2019;51(2):214–224. doi:10.1016/j.immuni.2019.08.003. 31433969

[62] Chazara O, Chang CS, Bruneau N, Benabdeljelil K, Fotsa JC, Kayang BB, Loukou NE, Osei-Amponsah R, Yapi-Gnaore V, Youssao IA, et al. Diversity and evolution of the highly polymorphic tandem repeat LEI0258 in the chicken MHC-B region. Immunogenetics. 2013;65(6):447–459. doi:10.1007/s00251-013-0697-6. 23529664

[63] Boudeau J, Glasser AL, Julien S, Colombel JF, Darfeuille‐Michaud A Inhibitory effect of probiotic Escherichia coli strain nissle 1917 on adhesion to and invasion of intestinal epithelial cells by adherent–invasive E. coli strains isolated from patients with Crohn’s disease. Aliment Pharmacol & Therapeut. 2003;18(1):45–56. doi: 10.1046/j.1365-2036.2003.01638.x. 12848625

[64] Gonzalez-Vallina R, Wang H, Zhan R, Berschneider HM, Lee RM, Davidson NO, Black DD. Lipoprotein and apolipoprotein secretion by a newborn piglet intestinal cell line (IPEC-1). Am J Physiol. 1996;271(2):G249–259. doi:10.1152/ajpgi.1996.271.2.G249. 8770040

[65] Beug H, von Kirchbach A, Doderlein G, Conscience JF, Graf T. Chicken hematopoietic cells transformed by seven strains of defective avian leukemia viruses display three distinct phenotypes of differentiation. Cell. 1979;18(2):375–390. doi:10.1016/0092-8674(79)90057-6. 227607

[66] Weingartl HM, Sabara M, Pasick J, van Moorlehem E, Babiuk L. Continuous porcine cell lines developed from alveolar macrophages: partial characterization and virus susceptibility. J Virol Methods. 2002;104(2):203–216. doi:10.1016/S0166-0934(02)00085-X. 12088830 PMC7119708

[67] Roche SM, Gracieux P, Milohanic E, Albert I, Virlogeux-Payant I, Temoin S, Grépinet O, Kerouanton A, Jacquet C, Cossart P, et al. Investigation of specific substitutions in virulence genes characterizing phenotypic groups of low-virulence field strains of *Listeria monocytogenes*. Appl Environ Microbiol. 2005;71(10):6039–6048. doi:10.1128/AEM.71.10.6039-6048.2005. 16204519 PMC1265998

[68] Bolger AM, Lohse M, Usadel B. Trimmomatic: a flexible trimmer for Illumina sequence data. Bioinformatics. 2014;30(15):2114–2120. doi: 10.1093/bioinformatics/btu170. 24695404 PMC4103590

[69] Escudié F, Auer L, Bernard M, Mariadassou M, Cauquil L, Vidal K, Maman S, Hernandez-Raquet G, Combes S, Pascal G. FROGS: find, rapidly, OTUs with galaxy solution. Bioinformatics. 2018;34(8):1287–1294. doi: 10.1093/bioinformatics/btx791. 29228191

[70] Mahé F, Rognes T, Quince C, de Vargas C, Dunthorn M Swarm: robust and fast clustering method for amplicon-based studies. PeerJ. 2014;25:2:e593. doi: 10.7717/peerj.593. PMC4178461 25276506

[71] Camacho C, Coulouris G, Avagyan V, Ma N, Papadopoulos J, Bealer K, Madden TL BLAST+: architecture and applications. BMC Bioinf. 2009;10:1–9. doi: 10.1186/1471-2105-10-421. PMC2803857 20003500

[72] Quast C, Pruesse E, Yilmaz P, Gerken J, Schweer T, Yarza P, Peplies J, Glöckner FO The SILVA ribosomal RNA gene database project: improved data processing and web-based tools. Nucleic Acids res. 2012;41(D1). doi: 10.1093/nar/gks1219. PMC3531112 23193283

[73] Oksanen J, Simpson GL, Blanchet FG, Kindt R, Legendre P, Minchin PR, Rb O, Solymos P, Stevens MH, Szoecs E, et al. vegan: Community ecology package (2.6-4) [Internet]. CRAN [cited 2022]; Available from https://CRAN.R-project.org/package=vegan.

[74] Love MI, Huber W, Anders S Moderated estimation of fold change and dispersion for rna-seq data with DESeq2. Genome Biol. 2014;15(12):550. doi: 10.1186/s13059-014-0550-8. 25516281 PMC4302049

[75] Pinheiro J, Bates D, Core Team R. Nlme: linear and nonlinear mixed effects models. R package version 3.1-157 [internet] CRAN [cited 2022]; Available from: https://CRAN.R-project.org/package=nlme.

[76] McMurdie PJ, Holmes S Phyloseq: an R package for reproducible interactive analysis and graphics of microbiome census data. PloS One. 2013;8(4). doi: 10.1371/journal.pone.0061217. PMC3632530 23630581

[77] Caspi R, Altman T, Billington R, Dreher K, Foerster H, Fulcher CA, Holland TA, Keseler IM, Kothari A, Kubo A, et al. The MetaCyc database of metabolic pathways and enzymes and the BioCyc collection of Pathway/Genome databases. Nucleic Acids Res. 2014;42(1). doi: 10.1093/nar/gkt1103. PMC3964957 24225315

[78] Ghazi AR, Sucipto K, Rahnavard A, Franzosa EA, McIver LJ, Lloyd-Price J, Schwager E, Weingart G, Moon YS, Morgan XC, et al. High-sensitivity pattern discovery in large, paired multi-omic datasets. Bioinformatics. 2022;38(Supplement_1). doi: 10.1093/bioinformatics/btac232. PMC9235493 35758795

