# Supplementary material for: The immune response modulated by inoculation of commensal bacteria at birth impacts the gut microbiota and prevents Salmonella colonization
Source: Gut Microbes. 2025 Mar 13;17(1):2474151. doi: 10.1080/19490976.2025.2474151 (PMC11913379; doi:10.1080/19490976.2025.2474151)
Supplement: Supplemental Material [file KGMI_A_2474151_SM9450.docx]

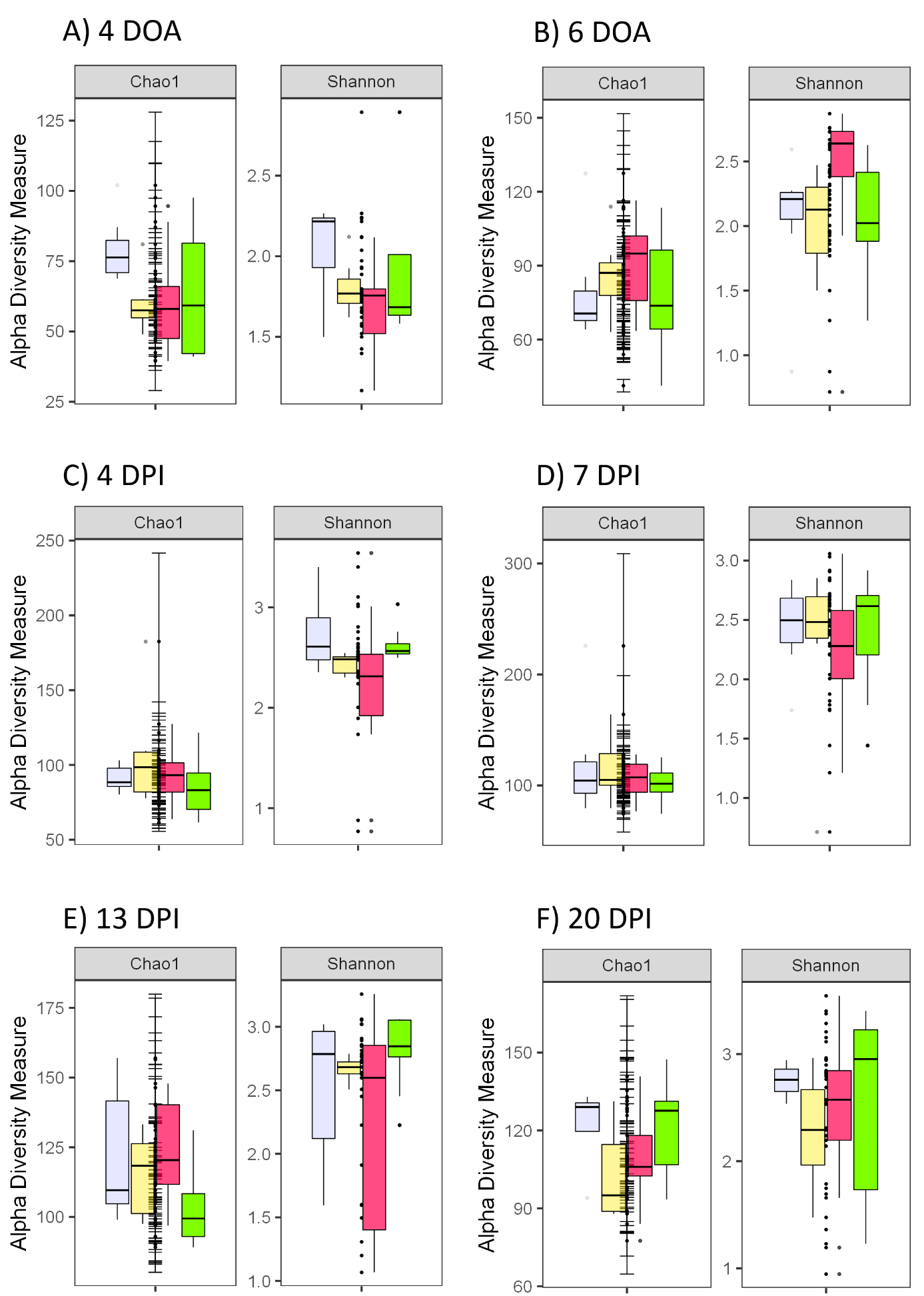
**Supplmentary Figure 1:** α-diversities (Chao1 and Shannon indexes) measured at the different timepoints for the four conditions ‘+Mix4’ (light blue), ‘Mix4 + SE’ (yellow), ‘+SE’ (pink) and ‘Ctrl’ (green).

**Supplementary Figure 2**: Kinetics of *Salmonella* colonization levels across the timepoints, for each chicken of the SLT-SS (green), SLT-LS (red) or ST-SS (blue) category, in the two groups infected with *Salmonella* at 7 DOA and treated or not with the MiX4 at hatch.

**Supplementary Table 1**: Main OTUs showing significant differential abundances between the SLT-SS and SLT-LS chickens after infection.

|  | |  | | log2FoldChange | | padj | | Family | | Genus | |
| --- | --- | --- | --- | --- | --- | --- | --- | --- | --- | --- | --- |
| 4 days p.i. | | Cluster_4 | | 7.08 | | 1.47x10^-11^ | | Lactobacillaceae | | *Pediococcus* | |
|  | | Cluster_114 | | 9.06 | | 1.93x10^-10^ | | Campylobacteraceae | | *Campylobacter* | |
|  | | Cluster_9 | | 7.19 | | 2.70x10^-9^ | | Clostridiaceae 1 | | *Clostridium sensu stricto 1* | |
|  | | Cluster_6 | | 4.94 | | 5.44x10^-5^ | | Enterococcaceae | | *Enterococcus* | |
|  | | Cluster_120 | | 6.54 | | 8.00x10^-4^ | | Micrococcaceae | | *Micrococcus* | |
|  | | Cluster_19 | | 5.50 | | 1.37x10^-3^ | | Lachnospiraceae | | *[Ruminococcus] torques group* | |
|  | | Cluster_41 | | 7.03 | | 5.42x10^-3^ | | Lachnospiraceae | | *Blautia* | |
|  | | Cluster_27 | | -6.33 | | 5.42x10^-3^ | | Ruminococcaceae | | *Oscillibacter* | |
|  | | Cluster_493 | | 5.54 | | 6.03x10^-3^ | | Enterobacteriaceae | | *Escherichia-Shigella* | |
|  | | Cluster_63 | | -3.49 | | 6.03x10^-3^ | | Enterobacteriaceae | | *Enterobacter* | |
|  | | Cluster_20 | | 3.87 | | 6.03x10^-3^ | | Clostridiaceae 1 | | *Clostridium sensu stricto 1* | |
|  | | Cluster_33 | | 5.80 | | 6.03x10^-3^ | | Clostridiaceae 1 | | *Clostridium sensu stricto 1* | |
|  | | Cluster_934 | | 6.94 | | 6.03x10^-3^ | | Lactobacillaceae | | Multi-affiliation | |
|  | | Cluster_37 | | 5.15 | | 6.55x10^-3^ | | Lachnospiraceae | | *Epulopiscium* | |
|  | | Cluster_46 | | -5.87 | | 7.51x10^-3^ | | Erysipelotrichaceae | | *Erysipelatoclostridium* | |
|  | | Cluster_79 | | 6.18 | | 8.93x10^-3^ | | Lactobacillaceae | | *Lactobacillus* | |
|  | | Cluster_150 | | 5.19 | | 8.99x10^-3^ | | Pseudomonadaceae | | *Pseudomonas* | |
| 7 days p.i. | | Cluster_27 | | -7.76 | | 0.0034 | | Ruminococcaceae | | *Oscillibacter* | |
|  | | Cluster_17 | | -8.66 | | 0.0034 | | Ruminococcaceae | | *Butyricicoccus* | |
|  | | Cluster_38 | | 4.91 | | 0.0039 | | Lachnospiraceae | | Unknown genus | |
|  | | Cluster_40 | | 5.08 | | 0.0039 | | Ruminococcaceae | | *DTU 089* | |
|  | | Cluster_4 | | 4.22 | | 0.0039 | | Lactobacillaceae | | *Pediococcus* | |
|  | | Cluster_2 | | -5.33 | | 0.0095 | | Enterobacteriaceae | | *Klebsiella* | |
|  | | Cluster_58 | | 5.59 | | 0.0095 | | Ruminococcaceae | | *Ruminococcaceae UCG-005* | |
| 13 days p.i. | | Cluster_17 | | -9.29 | | 1.58x10^-5^ | | Ruminococcaceae | | *Butyricicoccus* | |
|  | | Cluster_11 | | -4.00 | | 3.40x10^-5^ | | Lachnospiraceae | | Unknown genus | |
|  | | Cluster_7 | | -3.74 | | 3.40x10^-5^ | | Ruminococcaceae | | *Flavonifractor* | |
|  | | Cluster_2 | | -6.98 | | 1.38x10^-4^ | | Enterobacteriaceae | | *Klebsiella* | |
|  | | Cluster_1 | | 3.32 | | 8.87x10^-4^ | | Enterobacteriaceae | | *Escherichia-Shigella* | |
|  | | Cluster_14 | | -5.76 | | 9.43x10^-4^ | | Enterobacteriaceae | | *Klebsiella* | |
|  | | Cluster_34 | | -5.16 | | 1.46x10^-3^ | | Ruminococcaceae | | Multi-affiliation | |
|  | | Cluster_27 | | -6.06 | | 1.46x10^-3^ | | Ruminococcaceae | | *Oscillibacter* | |
|  | | Cluster_175 | | 5.33 | | 2.17x10^-3^ | | Enterobacteriaceae | | *Escherichia-Shigella* | |
|  | | Cluster_8 | | -2.74 | | 2.70x10^-3^ | | Lachnospiraceae | | *[Ruminococcus] torques group* | |
|  | | Cluster_51 | | -5.22 | | 2.70x10^-3^ | | Lachnospiraceae | | *CHKCI001* | |
|  | | Cluster_56 | | -2.56 | | 9.82x10^-3^ | | Lachnospiraceae | | *[Ruminococcus] gauvreauii group* | |
| 20 days p.i. | | *No differences* | | | | | | | | | |

Positive log2 fold changes correspond to the OTUs enriched in the SLT-LS chickens.

**Supplementary Table II:** Relative gene expression in blood of chicks infected with *Salmonella* Enteritidis and treated or not with the Mix4

| Gene | FC 4 DOA | FC 6 DOA | FC at 11 DoA | FC at 14 DoA | FC at 20 DoA | FC at 27 DoA |
| --- | --- | --- | --- | --- | --- | --- |
|  |  | | | | | |
| *Casp1* | 3.2 |  |  | 5.0 | 5.3 | 6.0 |
| *SOCS3* |  |  |  |  | -2.2 |  |
| *GAL2* |  |  |  |  |  | -2.0 |
| *HSP60* |  |  |  |  |  | -2.0 |
| *GAL6* |  |  |  |  |  | -2.1 |

FC represents the ratio between chicks treated with Mix4 and infected with *S.* Enteritidis and chicks infected with *S.* Enteritidis without presence of MIX4. Positive FC means that the gene is over-expressed in infected chicks without MIX 4. Ratio >2.0, with a p value <0.05 were considered

**Supplementary Table III:** Primers used with the Biomark to analyze the chicken response.

| **Gene** | **Forward primer** | **Reverse primer** |
| --- | --- | --- |
| *AH221* | TCTGCTCCTCCTGGCCCTCT | TCACCAGGATCACCGCTGG |
| *ALOX5AP* | CAGGCTCCTCTGCCTTTGAA | AAGCACGGCGAGAAACGTA |
| *ARG2* | GGCATGTACATTGCAGAGGAAA | GCTCCAAGCAGTGGATTGAC |
| *ARPC4* | GTGGAGCGCCACAACAAAC | TGATGATCACTGGCTGCAACA |
| *BF2* | AGGAGAGAGCGACCCGAGGT | ATGGGCACCAGGTTGGGCTG |
| *BIRC2* | GGGAGGAAGAGAAAGAGAGACA | ACGCTGGAATAAAGCCATCC |
| *BLB21* | AGAGGAGCGTGGAGCCCAAG | CAGCACCTGGTACGTCCAGT |
| *Casp1* | TATTCTTCCACGCCTGATACT | GAACTTCTTCAGCATTGTAGTC |
| *Casp8* | GGGTGTCTCCGTTCAGGTATC | GGCTCTTGTCCACTTTCCCA |
| *CCR2* | GGACAGTTACCTACGGCATCC | CCCCGGAACAGAGGCAAAA |
| *CCR6* | AAAGTGTTCCTGCCAGTTGC | CCGTCATGGACTTGGTTCGT |
| *CD14* | CCCTGCACCTGGACAACAAC | ACATCCTGCAGGTGCTCCA |
| *CD180* | CACCCGCATAGTGGATGCAA | ATCTGACACCTCGTCAGGTCCA |
| *CD25* | CCGAAGCAAGCAAACAATTCA | AAGATGACTGCCTCCATAAGAT |
| *CD28* | GTCAGAGAGACACCCATCCAA | AGTCTCGTGTTGGTGCATAGG |
| *CD40LG* | TGGTGGTACAGACCATTGGG | TGGCTGTCCTATCCTTGCATT |
| *CD47* | AAAACCTCCAACACCAGTGGATA | AACAGCAACACTGCGATGAC |
| *CD69* | TCATCATGCAGTACCACGGC | GCCTTGCGGCTTAAACCAGT |
| *CD80* | CACTTGCTTCGTTGAATATGAT | AAAACAGTGGGAACAGGCGT |
| *CEBPB* | AACGAGAGGCTGCAGAAGAA | CGGGCAGCTGTTTGAACAA |
| *CISH* | AGAAGATCCAAAGGCTGTCGCTG | AGCCCCAGTACCAGCCGGA |
| *CSF2RA* | TGGCAGTTGCAACACTCTTA | TGGCTTTGGGATTTGTGGAA |
| *CTLA4* | AAGGGAAATGGGACGCAACT | CTCTGAATCGCTTTGCCCAC |
| *CTSG* | TACGAACCAGAGCAGAGCCA | TTGACCTTCGCTGTCAGCTTG |
| *DDX3X* | TGGCTTACGAACAGCATCAC | TCTGGCACCAAATCCTCCA |
| *DEC205* | ATTCAGGCAAGTTGGGGCAG | TCCAAACGAAACCTCCGTGT |
| *GAL2* | CTGGTTTGGCTTTTGGGCTC | GCGAAGACAACCCTGGAGAA |
| *GAL6 / AvBD9* | GTTGCAGGTCAGCCCTACTT | TAGAGTGCCAGAGAGGCCAT |
| *GATA3* | TCGAGCACAGAAGGCAGGGA | GACAGTTAGCACAGGACGTG |
| *HMOX1* | ACGCTGAAAGCATGTCCCAG | AGATGAAGTACAGGGACGCC |
| *HSP60* | CAGAAGAAATTGCACAGGTTG | TGGAATTCACATTTCTGCCCT |
| *HSP90AA1* | ACACATGCCAACCGCATTTA | CCTCCTCAGCAGCAGTATCA |
| *IFNAR1* | AGCAGAGAGGAATGCATCGG | GCTCCGAAGCCCTCTATGTT |
| *IFNAR2* | GTAGCCACAGTAACTGGAAGA | CAGTGGACACAGGAACTGAGA |
| *IFNB* | TGACTGCAAACCATCAGTCTC | GGAGGTGGAGCCGTATTCTG |
| *IFNGR1* | TCGGCCTTGGATGAGAGAGA | TTGTGTACTCCAAGCCTGCG |
| *IGSF6* | GGAACGAAGGGCAGAGAACA | AGTTCTTGGGCTATTGCTCGAA |
| *IL10* | GTTTAAGGGGACCTTTGGCTG | TGCTCTGCTGATGACTGGTG |
| *IL12B* | AAGCTATAAAGAGCCAAGCAAGAC | GGTGCTCTTCGGCAAATGGA |
| *IL15* | GACTGGACTAACCATCTTCTTCC | CAGAACGTCTGACCACTTACA |
| *IL18* | CGTGGCAGCTTTTGAAGATGTA | CTGAATGCAACAGGCATCCC |
| *IRF2* | ACCGGATGCTGCCATTATCT | GCGGCTGTCCTACAACTACAA |
| *IRF7* | CACAACGCCAGGAAGGATGT | TGTCATTGGGGACGCCTGAG |
| *LEF1* | AACGACCCGTACATGCCTAA | AACTGCGTGAGAAGGCTGTA |
| *LGALS3* | TCGGCTGCTCATAACCATCA | TCGCAGAGCACCTGGAGCTTG |
| *LITAF* | ACAAGTACATCTGTTACAGTTC | AACCAGCTATGCACCCCAGC |
| *MDA5* | GGAAATACAGGAGGAACAGAGGA | GCTCTGTCCCAGGTTTTCGT |
| *MX1* | GGGGAACCAGCCACAAGATA | TTAGTGAGGACCCCAAGCGT |
| *MyD88* | CGTGCCAAAGACTTCAGAGC | ACCATCCTCCGACACCTTCTT |
| *NFKB1* | CGAACAGCAGATGGACCGTA | TTACCCACCAAGCTGTGAGC |
| *NLRC3* | AGCCCTGAAGGTCAATCACAG | GCTGCTAAGTCCAGCTTCCTCA |
| *NOD1* | AGCTTGCAGTGATCAGGCTC | TGCTCTTCTGAATGGCCTGG |
| *NOS2* | AATGAGTACCGTGTGCGAGG | TACAGCCTTGGCCAAAATGC |
| *NUMB* | TGGATGAATCCAGAGGAATGCA | GCTTTAACCGCCTTCTTCCC |
| *PGK1* | GCTAGAGAACCTCCGATTCCA | CCTCCACTTTTGCAGCATCA |
| *SDCBP* | TGGCAACAGCTGGGAATGTA | AGAGTGATCCATGAGGCTTTTCA |
| *SERPINB1* | ACGTCTACCATAAAGCAGAGAG | TATGGCTGCTTCTAGCTTCTC |
| *SIVA1* | CAGCTGCTGTAATGCTGTTACC | GCAGAGAACTTGCTCACCAAC |
| *SLC11A1* | CATGCATGACTTCGCCAAC | GTACACATTGATGGCACACAC |
| *SOCS1* | CACGCACTTCCGAACCTTTC | ACTTCAGCTTCTCATGGGCG |
| *SOCS3* | TAAGACTGTCAACGGGCACC | CCCCTGCTCTTGTGCTTGTA |
| *SPI1* | GACAGCCCCATGACTATTACCA | TGCACGTGGTGTGGATGATA |
| *STAT3* | AAGGGTGACCCAATTGTCCA | TGTTAAACTTCCGGGACCCCC |
| *STAT4* | CAATGAAGCGATGGCAACCA | ACTTTTCCAGTGGTCCCTGC |
| *STAT6* | GCGCAGTTCAATAAGGAGATC | GAAGCCAATGATCAGCCGGT |
| *TGFBR1* | CCCCCAACCACAGAGTAGGA | ACTCGTAATGCCTCACAGCTC |
| *TGFB3* | GGGCCCTGGATACCAACTAC | GGTCCTGTCGGAAGTCAATGTA |
| *TLR1* | TGAGCTTCATGACCAGCCGT | TGGTTGTTTTGTAGGTCCACT |
| *TLR15* | CGTACGTCCAGAAAACCCCA | CTGGAAGGCTTTCGATGGGT |
| *TLR2* | GCTCACAGGCAAAATCACGG | AGCGAAAAAAGGGGCCCAAAC |
| *TLR4* | CAGTCCGTGCCTGGAGGTCA | GTATGGATGTGGCACCTTGA |
| *TLR5* | TGTGTTGTGACCAGGCAGTT | AATCTTCAGGCCAACGCAGA |
| *TNFRSF1A* | ATGCAGTAGCCCAGTGACTA | GCCACAATGATGCCAAGAAC |
| *TREM2* | AGACCCAAATCCCAGAGGAAC | GCCACCACAAACTTAGTAGCC |
| *LDHA* | AGACTGGGCATCCATCCTCT | ATAGGCACTGTCCACCACCT |
| *SDHA* | CGTGATCTGGCTCATCTAAAGAC | TTGCAGTTCAAGGGTCTCCA |

**Supplementary Table IV:** Correlations between immune gene expression levels and taxon abundance

| **Days of age** | **SLT-LS** | **SLT-SS** | **ST-SS** |
| --- | --- | --- | --- |
| **4** | 13^#^ | 28 | 40 |
| **6** | 0 | 2 | 1 |
| **11** | 5 | 0 | 3 |
| **14** | 22 | 0 | 0 |
| **20** | 37 | 0 | 0 |
| **27** | 45 | 0 | 94 |

# These correlations were obtained using HAllA by analysis of significantly differentially immune gene expression levels (dCt) and taxon abundances
